# The genome of the Gulf pipefish enables understanding of evolutionary innovations

**DOI:** 10.1186/s13059-016-1126-6

**Published:** 2016-12-20

**Authors:** C. M. Small, S. Bassham, J. Catchen, A. Amores, A. M. Fuiten, R. S. Brown, A. G. Jones, W. A. Cresko

**Affiliations:** 1 Institute of Ecology and Evolution, University of Oregon, Eugene, OR 97403 USA; 2 Present address: Department of Animal Biology, University of Illinois at Urbana-Champaign, Urbana, IL 61801 USA; 3 Institute of Neuroscience, University of Oregon, Eugene, OR 97403 USA; 4 Present address: Oregon Health & Science University, Portland, OR 97239 USA; 5 Department of Biology, Texas A&M University, College Station, TX 77843 USA

**Keywords:** *Syngnathus scovelli*, Syngnathidae, Male pregnancy, Genome assembly, Evolution, Differential expression, Gene loss, Novel traits

## Abstract

**Background:**

Evolutionary origins of derived morphologies ultimately stem from changes in protein structure, gene regulation, and gene content. A well-assembled, annotated reference genome is a central resource for pursuing these molecular phenomena underlying phenotypic evolution. We explored the genome of the Gulf pipefish (*Syngnathus scovelli*), which belongs to family Syngnathidae (pipefishes, seahorses, and seadragons). These fishes have dramatically derived bodies and a remarkable novelty among vertebrates, the male brood pouch.

**Results:**

We produce a reference genome, condensed into chromosomes, for the Gulf pipefish. Gene losses and other changes have occurred in pipefish *hox* and *dlx* clusters and in the *tbx* and *pitx* gene families, candidate mechanisms for the evolution of syngnathid traits, including an elongated axis and the loss of ribs, pelvic fins, and teeth. We measure gene expression changes in pregnant versus non-pregnant brood pouch tissue and characterize the genomic organization of duplicated metalloprotease genes (*patristacins*) recruited into the function of this novel structure. Phylogenetic inference using ultraconserved sequences provides an alternative hypothesis for the relationship between orders Syngnathiformes and Scombriformes. Comparisons of chromosome structure among percomorphs show that chromosome number in a pipefish ancestor became reduced via chromosomal fusions.

**Conclusions:**

The collected findings from this first syngnathid reference genome open a window into the genomic underpinnings of highly derived morphologies, demonstrating that de novo production of high quality and useful reference genomes is within reach of even small research groups.

**Electronic supplementary material:**

The online version of this article (doi:10.1186/s13059-016-1126-6) contains supplementary material, which is available to authorized users.

## Background

Evolutionary novelties adorn the tree of life and yet their genetic origins remain a problem for biologists. The Modern Synthesis sparsely addressed novel traits but rationalized their incidence with neo-Darwinian models of gradual change via accumulation of many small-effect mutations [1]. Contemporary perspectives are more accepting of discontinuous morphological change [2], underlain by genetic changes diverse in nature. These changes may include point mutations as well as gross changes like gains and losses of genes or their regulatory elements, but the common thread is their effect on developmental systems. Indeed, the origin of novelties is now routinely viewed through the lens of evolutionary developmental biology, with an emphasis on how gene regulatory networks arise de novo or are modified from ancient ones [3] to orchestrate novel gene expression in development [4].

This modern genetic and developmental understanding of novel traits is an extremely difficult objective without quality genomic resources. Past genome sequencing efforts have been the purview of large, well-populated research communities generally focused on producing a resource beneficial for biomedical research. In the midst of the current sequencing technology revolution, however, the door is open for small research groups to produce genome resources for a variety of other questions, including those in ecology, conservation biology, evolutionary biology, and population genomics. As new evolutionary lineages are sampled, a valuable by-product is that novel reference genomes can augment the study of other existing model genomes, in the way the spotted gar (*Lepisosteus oculatus*) genome aids in bridging between the tetrapod and teleost model organisms [5]. We set out to genomically enable the study of novel body plan and reproductive character evolution in syngnathid fishes (pipefishes, seahorses, and seadragons) by generating a high-quality reference genome for the Gulf pipefish, *Syngnathus scovelli*.

**Fig. 1:**
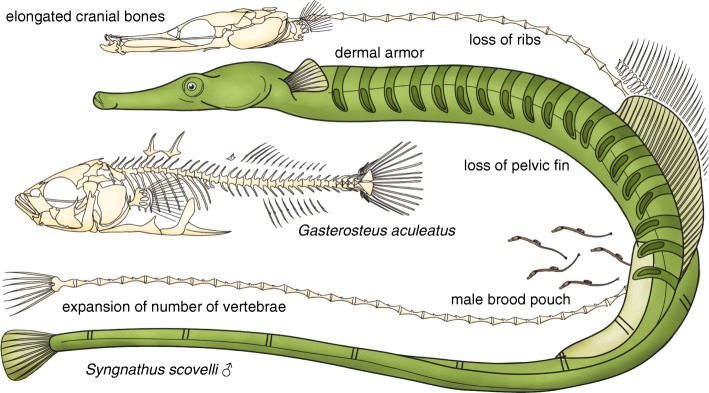
A cartoon representation of key derived traits in pipefishes and their relatives. Syngnathid fishes such as the Gulf pipefish have increased numbers of vertebrae and an elongated head, are missing pelvic fins and ribs, and have an evolutionarily novel structure, the male brood pouch. Shown for comparison is the axial skeleton of a percomorph with more typical morphology, a threespine stickleback. Note that not all derived syngnathid skeletal features are depicted in this cartoon. For detailed, anatomical illustrations of syngnathid skeleton attributes, please see other studies [144, 145]

Syngnathid fishes are widely recognized for their highly divergent body plans [6–8], including the elongate form of many pipefishes (Fig. 1), the upright body axis and reduced craniovertebral angle of seahorses, and the highly cryptic morphology of the seadragons. Derived characters such as leafy appendages, prehensile tails, and bony body armor are common across the family and, in many cases, have evolved independently in multiple lineages [6, 8, 9]. A truly striking evolutionary innovation shared by all syngnathid fishes is the somatic brooding of offspring by males, crowned by those lineages that have evolved complex, pouch-like structures for the maintenance of homeostasis during pregnancy [10–13]. In total, these remarkable characters make syngnathids an exceptional clade for the study of evolutionary novelty. The Gulf pipefish represents the group well, given its recent history as a choice subject for evolutionary genetic and behavioral studies [14–17], its abundance and amenability to experimental work, and its embodiment of many of the derived syngnathid traits.

Comparative genomics and evolutionary developmental approaches to effectively study the evolution of new forms, such as the diversification of the syngnathid body plan or the origin of male pregnancy, require advanced genomic tools. The centerpiece of each toolkit is a properly assembled, well annotated genome model, which can be directly compared at the sequence and structural levels to other species and efficiently mined to design molecular tools for manipulative genetic studies. To this end, we produced an annotated chromosome-level genome model [5] for *S. scovelli* by integrating a 176X-coverage, short-read genome assembly with a linkage map constructed from RAD-seq markers. We used this tool to reveal features of chromosome structure evolution, to investigate pipefish lineage-specific losses of genes associated with morphological development, to infer the likely phylogenetic position of the syngnathids in the tree of ray-finned fishes, and to describe a unique cluster of tandemly duplicated *patristacins* [18] that demonstrate conspicuous expression changes in the brood pouch during male pregnancy. Others have reviewed the approaches best suited to small-scale genome projects [19], but our intention here is to provide a biological case study and methodological template for success, motivated by the desire to better understand how novelties arise. We expect our experiences to be of interest to similarly sized research groups ready to reap the benefits of a reference genome in their own pursuits of biological discovery.

## Results

### The pipefish genome assembly is of high quality and completeness

The only published estimate of Gulf pipefish genome size is based on Feulgen staining [20], from which a haploid genome size of 523.23 Mb was calculated for the species. We obtained a short read k-mer-based genome length estimate of 351.44 Mb using ALLPATHS-LG [21]. Using the RAD markers from our genetic map to estimate the number of RAD sites per scaffold and infer the amount of sequence missing from the assembly by estimating the number of missing RAD sites, we obtained an estimated genome size of 334 Mb. These data suggest that, consistent with the k-mer-based estimate, no more than approximately 27 Mb, or 8% of sequence, is missing from the assembly (not including repetitive sequence) and that the Feulgen estimate is likely too large.

We assembled overlapping and mate-pair Illumina paired-end 100 nt reads (176X total coverage of 351 Mb) into 2123 scaffolds, yielding an assembly length of 307.02 Mb with 6.58% gaps. Contig and scaffold N50 were 32.24 kb and 640.41 kb, respectively, and the maximum scaffold size was 6.71 Mb. An analysis of core eukaryotic genes (CEGs) using CEGMA [22] revealed that our assembly contained complete information for 245 of 248 CEGs and “partial” information for the remaining three CEGs. These assembly quality metrics are comparable to other recently published, high-quality, scaffold-level genomes for fishes. Table 1 presents a side-by-side comparison of the Gulf pipefish assembly with several other published ray-finned fish assemblies.

**Table 1:** Scaffold-level assembly statistics for the Gulf pipefish genome

Genome	Scaffolds (n)	Longest scaffold	Scaffold N50	Contig N50	Assembly length	Gaps in assembly (%)	CEGs complete (%)
Gulf pipefish (*Syngnathus scovelli*)	2104	6.7 Mb	640.4 kb	32.2 kb	307.0 Mb	6.6	98.8
African turquoise killifish (*Nothobranchius furzeri*)	29,054	0.7 Mb	119.7 kb	8.7 kb	1010.9 Mb	7.7	94.8
Blind cave fish (*Astyanax mexicanus*)	10,542	9.8 Mb	1775.3 kb	14.7 kb	1191.1 Mb	19.1	87.9
Spotted gar (*Lepisosteus oculatus*)	2105	21.3 Mb	6928.1 kb	68.3 kb	945.8 Mb	8.1	90.7

The genome assembly of *S. scovelli* is comparable in quality to three recently published fish reference genomes. Shown in Table 1 are assembly statistics calculated from scaffold-level genome assemblies, considering scaffolds 1000 nt and longer, except for the 248-gene CEGMA analysis, which was applied to all scaffolds. Assembly versions are *N. furzeri* GCA_000878545.1 [23], *A. mexicanus* GCA_000372685.1 [24], and *L. oculatus* GCF_000242695.1 [5]

Using MAKER [25], we initially generated 37,696 total protein-coding gene annotations, but we retained only 20,834 of these based on biological evidence from protein databases, RNA-sequencing (RNA-seq) data, or protein domain detection. After manual annotation correction for several genes of interest, the final annotation included 20,841 protein-coding genes. Mean and median protein sequence length were 539.55 and 386.00 amino acids, respectively.

### A genetic map integrates 87% of the genome assembly into chromosomes

To order and orient scaffolds and to unite them into chromosomes, we generated an F1 pseudo-test cross genetic linkage map from a cross of wild *S. scovelli* with 108 progeny. Of 21,680 RAD tags, 4779 polymorphic tags were informative and met our criteria for inclusion in the genetic map (see “Methods”). The genetic map readily coalesced into 22 distinct linkage groups (see Additional file 1: Figure S1 for schematics of the consensus genetic map). Markers could be aligned to 553 scaffolds, thereby tying nearly 266.3 Mb – 87% – to chromosome models (see Additional file 2: SH1, which tabulates markers and scaffolds in the map). A total of 271 scaffolds (49%) were anchored at more than one map position with two or more markers, which allowed us to assign an orientation. Unplaced scaffolds tended to be shorter and more depauperate of annotated genes, on average, than scaffolds incorporated into chromosomes (see Additional file 1: Figure S2 for plotted lengths and gene densities of the scaffolds). Possibly the same sequence characteristics that make assembly difficult – a higher occurrence of repetitive DNA – could help explain the lower gene density of these smaller scaffolds. There were few initial conflicts between the genome assembly and the linkage map and none that could not be ruled out as artefactual due to poor support. For instance, three scaffolds were initially tied to more than one linkage group; in all three cases, however, only a single marker, with equivalent alignments to multiple locations, created this conflict and could be reasonably ruled incorrect, particularly when patterns of conserved synteny were taken into account. There were also apparent within-linkage group conflicts, which in most cases could be resolved by movement of markers without any cost to the linkage map. In total, five scaffolds where conflicts remained were split by our software Chromonomer (see “Methods”) to reconcile the map and the assembly; in each of these cases, a small scaffold (1.2 to 3.1 kb) was inserted into a gap in a larger scaffold. Only the largest of these small scaffolds contained an annotated gene, and in that case, its insertion into the larger scaffold agreed with the relative position of its ortholog in other teleost genomes.

### Chromosome evolution is revealed by patterns of conserved synteny

**Fig. 2:**
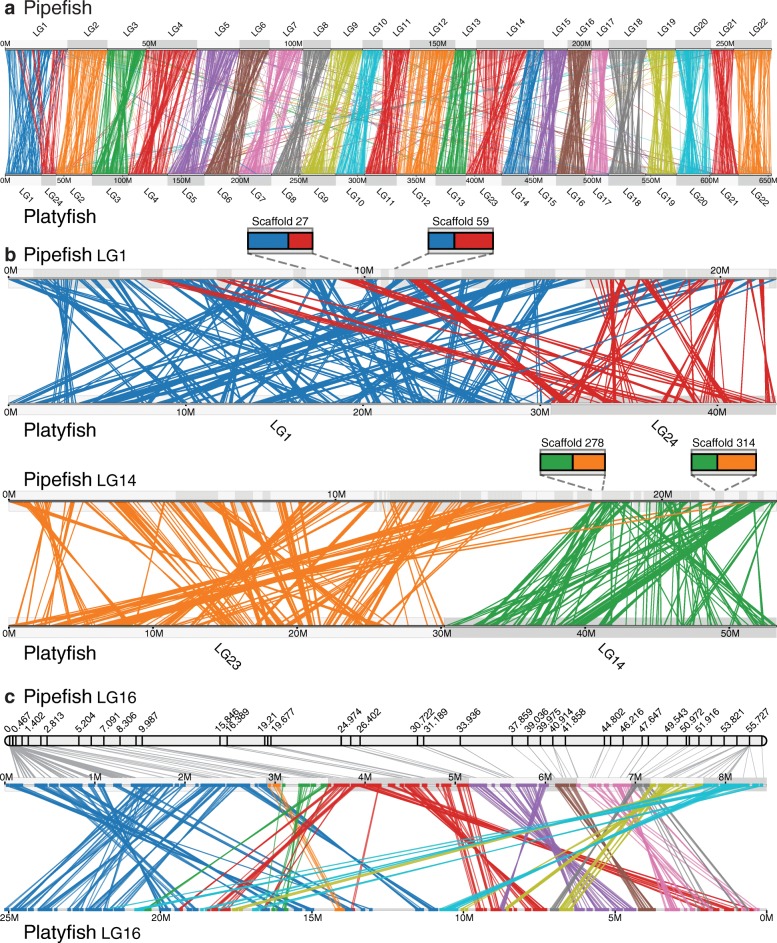
Chromosomal rearrangements inferred from a conserved synteny comparison. **a** Pipefish and platyfish chromosomes are broadly congruent. Strings connecting orthologous genes between the species’ genomes are colored by pipefish chromosome. **b** Pipefish LG 1 and 14 are each orthologous to two platyfish chromosomes, likely because chromosome fusions occurred in the syngnathid lineage. Several scaffolds from fused chromosomes 1 and from 14, including those shown in the insets, show blocks of conserved synteny to both “ancestral” chromosomes in platyfish (LG 1 and 24 or LG 14 and 23). This pattern indicates that some number of intra-chromosomal rearrangements blended segments across the chromosomal junction after the chromosomes fused. Strings connecting orthologs are color-coded by platyfish chromosome. Pipefish scaffolds are shown in alternately *shaded rectangles* along the chromosome. **c** On LG 16, differences in the orientation and location of orthologous gene blocks suggest inversions and transpositions have occurred since the last common ancestor of pipefish and platyfish. Strings connecting orthologous genes are colored according to the pipefish scaffold each gene resides on. Support for scaffold order and orientation can be seen in the linkage map for pipefish LG 16, shown above

Evidence based on ancestral state reconstruction supports an ancestral chromosome number of 24 in the teleosts [26]. Though chromosome number has been shown to vary across the broad group of Syngnathidae, the 22 linkage groups that coalesced in this linkage map in *S. scovelli* accords well with published karyotypes for two other species in *Syngnathus*, *S. abaster*, and *S. typhle* [27]. Using a genome-wide synteny analysis, we investigated how this change from the ancestral chromosome number likely occurred. Genes are called syntenic when they lie on the same chromosome or chromosomal segment and a pair of compared genomes show “conserved synteny” when orthologous genes that are syntenic in one genome also lie together, though not necessarily in the same gene order, in the comparator genome. The pattern of conserved synteny between Gulf pipefish and other teleosts, such as southern platyfish (*Xiphophorus maculatus*), which has the ancestral number of chromosomes (Fig. 2a), suggests that the reduced chromosome number in *Syngnathus* resulted simply from two chromosomal fusions (Fig. 2b). Two large blocks covering the length of one linkage group in *S. scovelli* have strong conserved synteny of orthologs along both platyfish LG 1 and 24, respectively, and another pair of blocks covering all of a second pipefish linkage group are orthologous to platyfish LG 14 and 23 (Fig. 2b). The resulting pipefish chromosomes, which we here name LG 1 and 14 to reflect this orthology, are the largest in the genome. Several scaffolds linked to pipefish LG1 and LG14 contain genes orthologous to the two ancestral chromosomes that constitute each of them (Fig. 2b), suggesting that intra-chromosomal rearrangements have blended the original margins of the chromosomes since they became fused.

Other within-chromosome rearrangements relative to various teleost reference genomes can be confidently inferred using the pipefish assembly and linkage map, where they provide mutual support. It is beyond the scope of this paper to catalogue such chromosomal differences and is the subject of other studies. As an example, however, pipefish LG 16 can be used to illustrate a subset of these rearrangements because all scaffolds that map to this linkage group are ordered and all but two very small scaffolds are oriented, with strong map support. Here, likely inversions and transpositions can be discerned in a comparison between pipefish and platyfish, based on stretches of conserved synteny of protein coding genes (Fig. 2c).

### Phylogenomic analysis supports an alternative hypothesis for the position of syngnathiform fishes among the Percomorpha

Knowing the phylogenetic placement of syngnathid fishes relative to other teleosts with sequenced genomes is critical for using comparative genomic approaches to polarize the evolution of traits in the Syngnathidae. Conflicting hypotheses regarding the origin of syngnathid fishes and their relatives are a barrier to this understanding, and resolving phylogenetic relationships for the crown clade of teleosts (Superorder Percomorpha) in general has been a problem [28–30].

**Fig. 3:**
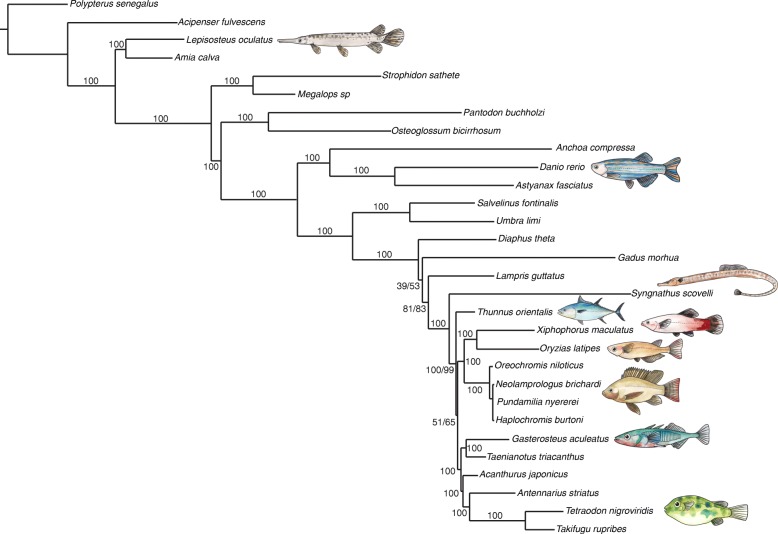
Phylogenomic inference supports a syngnathiform clade distinct from the clade containing commonly studied fish models. A well-supported maximum likelihood tree of UCEs places Syngnathiformes as an outgroup relative to fellow percomorph species used as genetic models, consistent with previous work regarding the molecular systematics of Percomorpha [29, 30, 33]. Note, however, that our topology is not consistent with a monophyletic group including Syngnathiformes and Scombriformes, as previously reported. Bootstrap and SH-aLRT support is listed for each node; a single number is listed where both values agree

Ultraconserved elements (UCEs) offer a genome-wide alternative to small panels of nuclear and mitochondrial phylogenetic markers because they exist by the hundreds or thousands in vertebrate genomes, are often easily identifiable as well-conserved, single-copy orthologs that contain divergent regions, and can be used to address hypotheses over a broad range of phylogenetic scales [31]. Faircloth et al. [32] used UCEs to produce a well-supported phylogeny at both deep and shallow time scales for ray-finned fishes. We added to this dataset UCEs from Gulf pipefish, Pacific bluefin tuna (*Thunnus orientalis*), and southern platyfish and performed phylogenetic analysis. Interestingly, our phylogenomic analysis provides an alternative hypothesis regarding the relationships among Scombriformes (tunas and their relatives) and Syngnathiformes (Syngnathid fishes and their relatives). Briefly, the two orders would not be interpreted as a monophyletic clade from our topology, in contrast to conclusions based on trees inferred by others [29, 30, 33]. Statistical support for clades bracketing this region of the topology was high (Fig. 3), but should be interpreted with caution given evidence that phylogenetic discordance across different regions of the genome can limit the accuracy of species-level inferences based on concatenated sequence data [34, 35]. We recovered all relationships reported by Faircloth et al. [32] and found, consistent with previous studies [29, 30, 33], that the Syngnathiformes are not nested within the clade containing species commonly used in genetic and genomic studies (i.e. medaka, platyfish, stickleback, and pufferfish). Given this phylogenetic hypothesis for the origin of syngnathids, the Gulf pipefish genome fills a useful outgroup role in comparative genomics studies using these model species. The currently understood relationships also highlight a need for phylogenetic analyses including fish lineages that diverged just prior to origin of the syngnathids, in order to help understand the unusual derived traits in the Syngnathidae.

### Convergent and unique gene losses have occurred in the pipefish *hox* clusters

**Fig. 4:**
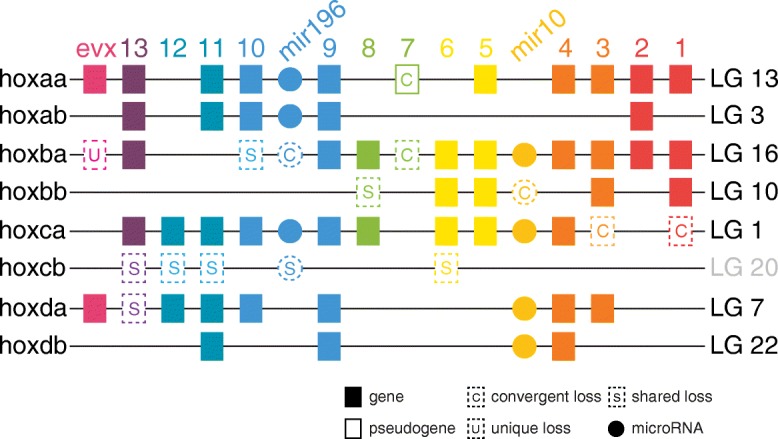
The pipefish *hox* clusters have experienced convergent and unique gene losses. A cartoon of the hox clusters in *S. scovelli*, with boxes representing genes arranged along chromosome segments of different linkage groups, summarizes gene content changes relative to other teleosts. Seven gene losses, of both coding and non-coding genes, are here labeled shared losses among the compared percomorph lineages because these genes are retained by the non-percomorph outgroup, zebrafish. Six other pipefish gene losses are inferred to be convergent losses with respect to some members of Percomorpha because other species that are not pipefish sister lineages have also lost these genes. *hox* cluster-associated *evenskipped* gene *eve1* (a member of the *evx* paralogy group) is missing in pipefish, a loss that has not been reported in other teleosts. Though percomorphs likely share the loss of the *hoxcb* cluster, comparison via conserved synteny with zebrafish shows that the orthologous region is on pipefish LG 20

The *hox* clusters, which include tandem arrays of homeobox genes interspersed with non-coding RNAs that regulate *hox* and other genes, are critical for patterning the body axis and paired appendages (reviewed in [36–38]). Pipefish have elongated bodies, including more trunk and especially more caudal vertebrae than relatives like medaka and threespine stickleback, and they lack pelvic fins, key examples of derived traits depicted in cartoon form in Fig. 1. We therefore scrutinized the gene content of the *hox* clusters for differences from pipefish’s percomorph relatives (including pufferfish, medaka, stickleback, and tuna). Just as in many other gene families, differential loss of *hox* genes among lineages followed the whole genome duplication that occurred near the base of the teleost lineage (e.g. [39]). Gulf pipefish appears to share some of these losses with other percomorph fishes, to the exclusion of the outgroup lineage zebrafish (Fig. 4). A parsimonious interpretation of the pattern of losses suggests that *hoxb10a*, *hoxb8b*, *hoxd13a*, the entire *hoxcb* cluster, and *mir196c* were absent in the common ancestor of pipefish and other percomorphs. Several other *hox* cluster genes have been lost in pipefish as well as in some but not all model percomorphs; based on the topology of the phylogenetic tree in Fig. 3 and those inferred by others [29, 30, 33], we conclude that these losses are likely to be convergent (Fig. 4). These include *hoxa7a*, *hoxb7a*, *hoxc3a*, *hoxc1a*, *mir196b* in the *hoxba* cluster, and *mir10a* in the *hoxbb* cluster. For example, *hoxb7a* was likely lost independently at least three times (in pufferfish, medaka, and pipefish), but it is still present in stickleback and tuna. *hoxa7a* was lost independently in both pipefish and pufferfish, leaving both lineages with no *hox7* paralog in any cluster. By contrast, zebrafish and all of the other percomorphs surveyed here retain either *hoxa7a* or *hoxb7a* or they have both of these genes. There is a remnant of the pipefish *hoxa7a* sequence, found between *hoxa5a* and *hoxa9a*; it is likely a pseudogene, as there is no trace of the sequence for the homeobox-containing second exon and an early stop codon in the first exon is predicted also to eliminate the hexapeptide. In addition to these losses, the pipefish *hoxba* cluster remarkably no longer has *evenskipped* gene *eve1*, a gene that is present in zebrafish and all other percomorphs compared here (Fig. 4). We detected pipefish sequences for orthologs of long non-coding RNA genes *hotairm1* between *hoxa1a* and *hoxa2a*, and *hottip* between *evx1* and *hoxa13a* (not shown). *hotairm1* is missing in zebrafish and so far unreported in any teleost (though annotated in the Ensembl reference genome for spotted gar, an actinopterygiian basal to the teleosts).

### *Syngnathus scovelli dlx* gene clusters are missing deeply conserved non-coding elements

**Fig. 5:**
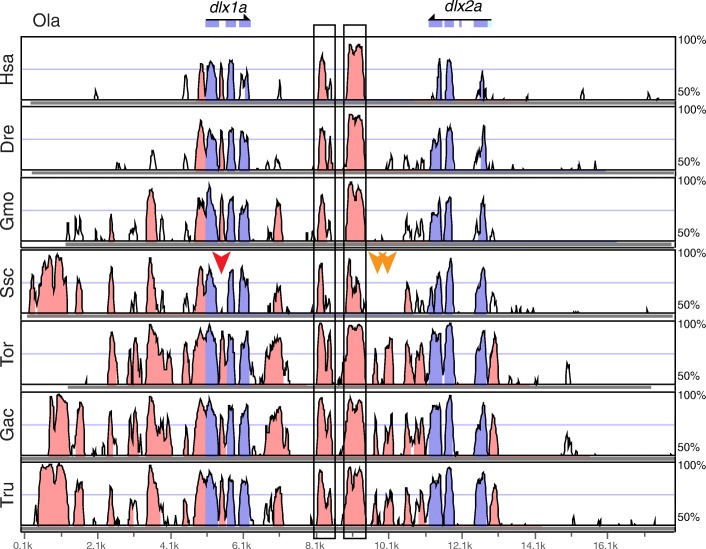
Three CNEs are not detectable in the pipefish *dlx1a-dlx2a* cluster. One CNE present in other teleosts and mammals is missing from a gapless region between exon1 and -2 in the *S. scovelli* assembly (*red arrow*). Two other CNEs in the *dlx* intergenic region that are conserved among percomorphs are also missing from this region in pipefish (*orange arrows*). Two CNEs previously shown to direct reporter gene expression in murine *Dlx* expression domains are boxed [43]. Exons are highlighted in *blue*, CNEs in *pink*. The reference, Ola, is medaka; Hsa, human; Dre, zebrafish; Gmo, cod; Ssc, pipefish; Tor, tuna; Gac, stickleback; Tru, pufferfish

The vertebrate *dlx* genes, a family of homeobox transcription factors important for patterning the central nervous system, head skeleton, and limbs, are arranged in tandem pairs associated with specific *hox* clusters. Some percomorphs, like stickleback and pufferfish, retain *dlx1/2a*, *dlx3/4a*, *dlx3/4b*, and *dlx5/6a* clusters, while medaka appears to lack a *dlx3/4a* cluster, and zebrafish (a non-percomorph) has lost *dlx3a* but has retained an unpaired *dlx2b* not found in percomorphs [40]. We found the four typical percomorph clusters, totaling eight genes, in the Gulf pipefish genome and performed a search via mVISTA [41, 42] for conserved non-coding elements (CNEs) within the *dlx* clusters by comparing sequences from mammals and other teleosts. We found that pipefish retains some non-coding elements conserved between mammals and teleosts, as well as other CNEs shared only among teleosts [40, 43] (Fig. 5; see Additional file 1: Figure S3 for VISTA comparisons of the *dlx3/4a*, *dlx3/4b*, and *dlx5/6a* clusters). For example, we identified pipefish orthologs of two inter-*dlx* CNEs (Fig. 5) that were found previously to be conserved between mouse, zebrafish, and pufferfish and that were shown to direct reporter gene expression in subsets of *dlx* domains [43]. A third CNE that was not functionally tested but was conserved in both zebrafish and pufferfish [43] is not preserved in pipefish. We identified two other notable losses in this pipefish cluster: *S. scovelli* has lost an inter-*dlx1/2a* CNE that we find conserved in the other percomorphs, and it also lacks an element in the intron between coding exon 1 and exon 2 of *dlx1a*, a CNE that is conserved in both mammals and other teleosts. There are no gaps in the assembly in these regions of the pipefish genome. Several other CNEs are missing from other clusters, including two elements on either side of the last exon of *dlx4a* that are, notably, conserved between other percomorphs such as pufferfish and stickleback and cod, a non-percomorph (Additional file 1: Figure S3).

### Syngnathid hindlimb loss implicates modification of the *tbx4-pitx1* pathway

**Fig. 6:**
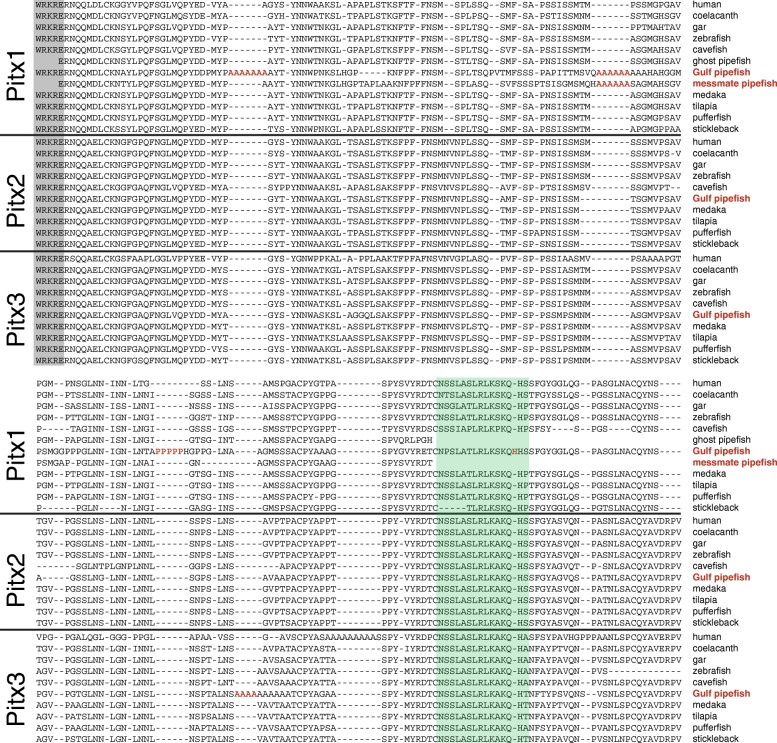
Pipefish Pitx1, a vertebrate protein important for hindlimb and tooth development, contains several homopolymeric expansions. Shown are well-aligned regions of Pitx proteins across several vertebrate species, starting from the last five amino acids of the homeodomain (shaded *gray*). Poly-alanine and poly-proline expansions (shown in *red*) in pipefish Pitx1 and Pitx3 between the homoedomain and the OAR domain (shaded *turquoise*) are not found in the Pitx proteins of other compared fish; however, there is a poly-alanine expansion at a different location in human Pitx3. One of the Pitx1 polyalanine expansions is shared with the messmate pipefish (*Corythoichthys haematopterus*), a distantly related syngnathid [11], and none are present in the robust ghost pipefish (*Solenostomus cyanopterus*), a member of a close, pelvic-fin-bearing outgroup to the syngnathids [72, 73]. Gulf pipefish also has a single amino acid insertion (also shown in *red*) in the conserved OAR domain

Pipefish, seahorses, and seadragons all lack paired pelvic fins. *tbx4*, *pitx1*, *and pitx2* are genes at the top of the regulatory cascade described in vertebrate hindlimb development, including teleosts that have pelvic fins [44–46]. We found no trace of the protein-coding sequence for *tbx4* in the pipefish genome assembly. The genomic segments flanking *tbx4* were also not identified, as pipefish orthologs of genes adjacent to *tbx4* in other teleosts were either undetected, as in the case of *tbx2b*, or were on small scaffolds not anchored to the genetic map. TBLASTN also failed to identify *tbx4* among our de novo assembled gene transcripts generated from RNA-seq data. Gulf pipefish *pitx1* is present in the assembly but divergent. The predicted pipefish Pitx1 amino acid sequence, supported by transcriptome sequencing, contains homopolymeric expansions of alanine and proline, and an amino acid insertion in the conserved OAR domain not seen in orthologs from other fish lineages or from human (Fig. 6). A fragment amplified with degenerate polymerase chain reaction (PCR) primers shows that a second syngnathid species, the messmate pipefish (*Corythoichthys haematopterus*), shares one of the alanine expansions (Fig. 6). Both Gulf pipefish and human Pitx3, a protein associated more strongly with eye and neural development than limb development [47, 48] also have polyalanine runs in different locations from those found in Pitx1. Pitx2 aligns well with other fish orthologs and apparently contains no homopolymeric expansions.

### Pregnancy-specific gene expression in the brood pouch is widespread and reflects regulation of the innate immune system

We aligned to the annotated genome RNA-seq data from six pregnant male brood pouches (excluding embryonic tissue) and six non-pregnant male pouches. Based on these digital gene expression data, the transcriptional landscape of male brooding tissues differed substantially as a consequence of pregnancy, as 26.19% of the total multivariate dissimilarity among the 12 individual transcriptomes was explained by pregnancy status (Additional file 1: Figure S4a; perMANOVA: *F*_1,11_ = 3.55, *p* = 0.004). Univariate tests of differential expression between pregnant and non-pregnant males revealed different transcript abundances for 1145 genes of 15,253 genes (false discovery rate (FDR) = 0.1) expressed robustly across at least four of 12 individuals. In total, 526 genes were pregnancy-enriched and 619 were pregnancy-depressed, demonstrating fold change differences as extreme as 215 (Tables 2 and 3; see Additional file 2: SH2 for a complete tabulation of differentially expressed genes).

**Table 2:** List of the top 15 pregnancy-enriched pouch tissue genes

Gene ID	Fold change	CPM	*p* value	Gene description	KO ID
SSCG00000006913	15.66	7.22	2.13E-24	WNT1-inducible-signaling pathway protein 2 isoform X2	K06827
SSCG00000005974	21.04	6869.88	1.87E-18	patristacin, partial	K08778
SSCG00000007802	4.15	93.44	7.69E-16	podocan	
SSCG00000014514	3.15	46.38	1.45E-15	fos-related antigen 2-like	
SSCG00000015977	12.38	229.24	1.39E-14	myocilin-like	
SSCG00000006209	6.53	4.72	4.91E-14	dickkopf-related protein 2	K02165
SSCG00000007875	2.93	188.72	8.81E-14	neuroepithelial cell-transforming gene 1 protein	
SSCG00000013720	5.13	233.89	3.85E-13	lipopolysaccharide-binding protein/bactericidal permeability-increasing protein	
SSCG00000011252	2.88	72.11	2.72E-12	beta-galactoside alpha-2,6-sialyltransferase 1-like isoform X1	K00778
SSCG00000004944	6.64	29.73	7.33E-12	collagen alpha-2(VI) chain-like	K06238
SSCG00000006480	3.10	18.93	1.81E-11	CTTNBP2 N-terminal-like protein	
SSCG00000013244	2.30	34.04	2.10E-11	LIM domain transcription factor LMO4-B-like	
SSCG00000004636	3.22	386.88	3.62E-11	NA	
SSCG00000002072	29.24	1.59	3.77E-11	potassium channel subfamily K member 2-like	K04913
SSCG00000007792	5.21	7.06	4.20E-11	excitatory amino acid transporter 5-like	K05618

Included are the fold change (pregnant/non-pregnant), average expression level across 12 pouch libraries in copies per million (CPM), edgeR negative binomial exact test *p* value, gene description from top BLASTP hit, and the assigned KEGG orthology ID for each pipefish gene. See Additional file 2 SH2 for the full list

**Table 3:** List of the top 15 pregnancy-depressed pouch tissue genes

Gene ID	Fold change	CPM	*p* value	Gene description	KO ID
SSCG00000006879	27.36	56.49	7.91E-43	Serine/threonine-protein kinase WNK2	K08867
SSCG00000018539	12.37	15.96	2.04E-26	FXYD domain-containing ion transport regulator 12	
SSCG00000007973	4.73	53.34	1.66E-24	A disintegrin and metalloproteinase with thrombospondin motifs 6, partial	K08621
SSCG00000013585	10.78	19.10	1.07E-23	Tetratricopeptide repeat protein 18	
SSCG00000005985	214.58	652.27	7.29E-23	patristacin, partial	K08076
SSCG00000008728	14.12	6.03	2.22E-22	Uridine-cytidine kinase-like 1	K00876
SSCG00000000969	4.32	19.82	1.25E-17	ras-like protein family member 11A	K07852
SSCG00000017729	6.14	359.52	1.71E-17	nidogen-2-like isoform X5	K06826
SSCG00000004506	6.00	12.98	4.08E-17	syntaxin-2-like isoform X1	K08486
SSCG00000010275	14.47	3.28	1.00E-16	acid-sensing ion channel 1	
SSCG00000016046	6.75	8.51	1.51E-16	leucine-rich repeat-containing protein 4-like	K16351
SSCG00000014649	10.15	7.67	1.77E-16	homeobox protein MSX-2-like	K09341
SSCG00000019217	66.66	3.26	1.82E-16	leucine-rich repeat-containing protein 3-like	
SSCG00000007661	5.19	24.20	2.23E-16	cytochrome P450 27C1-like	K17951
SSCG00000005388	19.81	1.44	5.60E-16	glutamate receptor ionotropic, delta-2 isoform X5	K05207

Included are the fold change (non-pregnant/pregnant), average expression level across 12 pouch libraries in copies per million (CPM), edgeR negative binomial exact test *p* value, gene description from top BLASTP hit, and the assigned KEGG orthology ID for each pipefish gene. See Additional file 2 SH2 for the full list

We identified several KEGG pathways enriched for genes subject to strong pregnancy-specific expression patterns, including “complement and coagulation cascades,” “cytokine-cytokine receptor interaction,” “calcium signaling,” and “neuroactive ligand-receptor interaction” (See Additional file 2: SH3 for a full tabulation of KEGG pathways enriched for differentially expressed genes). Many pipefish genes within the first two of these pathways, which include innate immune system cascades, were expressed at higher levels in pregnant, relative to non-pregnant, pouch tissues. For example, members of the complement membrane attack complex (MAC), which are cell membrane pore-forming toxins [49] (reviewed in [50]), tended to be expressed at higher levels in pregnant males (Additional file 1: Figure S5a, S6a). Pro-inflammatory chemokines Il8, Cxcl9, Cxcl10, and Cxcl12 of the Cxc subfamily were also expressed at higher levels in pregnant males, as were several members of the Cc subfamily (Additional file 1: Figure S5b). Not all transcriptional signatures of the immune system reflected this pattern, however. A suite of genes belonging to the natural killer cell cytotoxicity response pathway, for example, was expressed at higher levels in non-pregnant males (Additional file 1: Figure S4d). Furthermore, genes in KEGG pathways associated with the adaptive immune system, including “antigen processing and presentation,” “T cell receptor signaling pathway,” and “B cell receptor signaling pathway,” were transcriptionally less sensitive to pregnancy status than those in innate immunity KEGG pathways (Additional file 1: Figure S6b). Consistent with a characterization of the immune gene repertoire in *Syngnathus typhle* [51], we failed to detect MHC class II alpha and beta chain genes in the genome of *S. scovelli*, so the potential for some functionality of the adaptive immune system in this pipefish genus may be limited in general.

Gene Ontology terms overrepresented among pregnancy-enriched genes included those related to the complement system, coagulation, and immunity, consistent with the KEGG analysis, but we also identified terms related to hemopoiesis, homeostasis, proteolysis, and others (Additional file 2: SH5). GO terms overrepresented among pregnancy-depressed genes included those related to developmental processes, cell-to-extracellular matrix (ECM) adhesion, and protein glycosylation (Additional file 2: SH6).

### Lineage-specific duplication of *patristacins* associated with male pregnancy

**Fig. 7:**
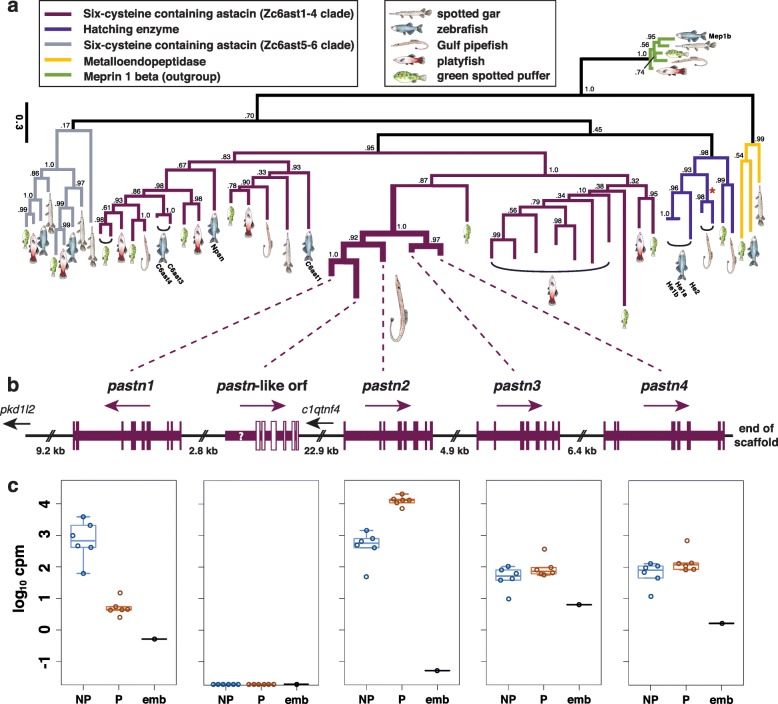
Gene duplication of *patristacins* preceded the evolution of diverse expression patterns related to male pregnancy. Patristacins are unique, tandemly arrayed C6 astacin-like metalloprotease genes presumably co-opted during the evolution of male pregnancy [18]. **a** A maximum likelihood gene tree inferred from astacin-like metalloprotease amino acid sequences, representing five fish genomes, is rooted assuming Meprin1b proteins as an outgroup. Different protein subfamily clades (colored by clade and including terminology from Kawaguchi et al. [89]) mostly correspond to conserved syntenic regions. Clade support values are SH-aLRT, but see Figure S8 (in Additional file 1) for bootstrap values and tip accession numbers. Zebrafish sequences with annotated Ensembl gene names are labeled for reference. Patristacins comprise a monophyletic group nested within the Zc6ast1-4 clade, suggesting pipefish or syngnathid lineage-specific duplication events. Note the absence of pipefish orthologs from the Zc6ast5-6 clade (colored *gray*). In medaka, orthologs from this group are expressed exclusively in the developing jaw [89]. Also note the *red asterisk* in the hatching enzyme clade, which corresponds to intron loss in the pipefish lineage. **b** The physical arrangement of *patristacins* in the Gulf pipefish genome, with two other genes in the region (*small text*). *Arrows* indicate the direction of the sense strand and *vertical bars* reflect coding exons. Note that the status of "*pastn*-like orf" as a gene is uncertain, so it is depicted by *open bars* and a *question mark* where three missing exons would normally be. **c** *Patristacin* expression levels from RNA-seq data for six non-pregnant male brood pouch samples (*blue*), six pregnant pouch samples not including embryos (*orange*), and a pooled embryo library (*black*). Y-axis values are copies per million (cpm) on a log scale. Individual *data points* and *boxplots* are shown. Note the extreme expression differences between *pastn1* and *pastn2*

As documented previously in *S. scovelli* and *S. floridae* [52], two similar astacin-like metalloproteases demonstrated strikingly opposite patterns of gene expression: one markedly pregnancy-enriched and the other highly pregnancy-depressed (Table 2, Table 3, Fig. 7b, c). We here find that these “*patristacins*” [18] are adjacent genes belonging to a small cluster of duplicates that includes two additional *patristacins* expressed at lower levels in the brooding tissues at the stages examined (Fig. 7b, c). This cluster, located on scaffold 62 of pipefish LG4, also included a fifth, partial coding sequence for which we could identify neither a likely start methionine nor the first three typical *patristacin* exons. A phylogenetic analysis including astacin-like metalloprotease sequences from global searches of five ray-finned fish genomes suggests that the *patristacin* cluster is a gene family expansion unique to the lineage leading to syngnathids (Fig. 7a). We found protein-coding genes from platyfish and green spotted puffer genomes that share a recent common ancestor with *patristacins*, but these sequences were not nested within the *patristacin* subclade. Furthermore, *patristacins* and their closest homologs most likely diverged via gene duplication from the subfamily of 6-cysteine astacins that includes zebrafish *nephrosin*, given the topology of our current gene tree and that all paralogs share the same genomic region on pipefish LG4.

## Discussion

Despite the explosive teleost species radiation over the last 300 million years, these fishes have been conservative in karyotype evolution relative even to the much younger mammalian lineage, with the majority of teleost species having a haploid number of 24 or 25 [53]. Variations from the inferred ancestral number of 24 [26] do exist across the teleost radiation, stemming from chromosome duplications, fissions, and fusions. We have shown that two chromosomal fusions in an ancestor of *Syngnathus scovelli* have likely led to a haploid karyotype of 22 (Fig. 2a and b). Comparisons of sequenced genomes suggest that interchromosomal rearrangements (translocations) are relatively uncommon in teleosts [53] and this is reflected in the striking one-to-one correspondence of chromosomes across most of the genome between Gulf pipefish and other percomorphs, such as southern platyfish (Fig. 2a). The stability of teleost genomes simplifies comparisons and increases confidence in correctly determining orthology of genes and chromosome segments based on observed patterns of conserved synteny. We have exploited the exceptional conservation of synteny among sequenced teleosts to explore the evolution and behavior of genes that might play a role in syngnathid innovations.

The remarkable morphology of syngnathids was noted in “The History of Animals” by Aristotle, who construed the peculiar phenomenon of pipefish live birth as a splitting open of the body. Prior to our characterization of the Gulf pipefish genome, however, with the exception of a few transcriptomic resources [51, 52, 54], virtually no information existed for how key developmental genes and their modification might be responsible for derived syngnathid phenotypes. Now, with the availability of the genome of *Syngnathus scovelli*, and likely other related genomes soon to follow, we expect researchers interested in the developmental genetic underpinnings of novel vertebrate morphologies to make the critical experimental connections between genomic differences in syngnathids and their functional consequences. In anticipation of exciting functional genomics work enabled by the latest genome editing approaches [55, 56], here we highlight a few especially promising examples of molecular signatures with implications for hallmark traits of pipefishes, seahorses, and their relatives.

We explored the constitution of the syngnathid *hox* genes because these Vertebrate Hox clusters are tandem arrays of transcription factor genes with many developmental roles, including segmental identity in the axis and in limb morphogenesis (reviewed in [36, 57]). Our investigation of Gulf pipefish *hox* cluster content revealed that the evolution of an elongated, ribless body was not accompanied by drastic reorganization of the *hox* genes. While there are multiple losses of pipefish *hox* genes and the *hox*-regulating microRNA genes that are interspersed among them, many of these same genes have been lost from other percomorphs that have less modified skeletons (Fig. 4).

Two gene losses from the Gulf pipefish hox clusters stand out, however. The loss of *eve1* is unique among described teleost *hox* clusters. This gene belongs to the *evenskipped* (*evx*) gene family, whose members reside at the ends of particular clusters. In zebrafish embryogenesis, the *hoxba* cluster-associated *eve1* gene is expressed during gastrulation and in the extending tail tip; its knockdown suppresses trunk and tail development, prompting the experimentalists to suggest *eve1* acts as a posterior organizer [58] (but see [59] for another interpretation). It is therefore remarkable that *eve1* is deleted in pipefish (Fig. 4). It is possible that some of these early ontogenetic functions of *eve1* have been distributed to the remaining two pipefish *evx* genes or otherwise compensated for. However, syngnathids have neither oral nor pharyngeal teeth, consistent with evolutionary loss of *eve1*, the only reported *evx* gene that is expressed during teleost tooth development [60, 61]. In addition, it appears that pufferfish and pipefish lineages have independently lost all copies of *hox7*, a paralogy group that when experimentally knocked out in mouse causes reduction and mispatterning of ribs [62]; consistent with this biological role for *hox7*, both pufferfish and pipefish lack ribs.

A uniting trait of the Syngnathidae is an absence of pelvic fins. Two other percomorphs that have evolutionarily lost pelvic fins appear to have done so by alteration of a hindlimb-positioning *hoxd9a* expression boundary (pufferfish [63]) or by loss of *pitx1* expression in the developing hindlimb (freshwater threespine stickleback [64, 65]). Pitx1, a transcription factor, directly activates initial expression of *tbx4* in the hindlimb primordium [66] and *tbx4* is required for initial limb bud outgrowth [67]. We found that pipefish *pitx1* has an amino acid insertion in the OAR, a functional domain thought to modulate DNA binding [68], and unusual homopolymeric alanine and proline repeat expansions between the homeodomain and OAR (Fig. 6). Homopolymers are known to cause several developmental diseases in humans (reviewed in [69]) and to affect subcellular localization, protein-protein interaction, and transcriptional regulation [70, 71]. In particular, expansions of alanine and proline homopolymers within transcription factors can modulate the proteins’ ability to regulate transcription of gene targets. A distantly related pipefish species, the messmate pipefish, shares one of the homopolymeric repeats (Fig. 6), suggesting that this divergence of *pitx1* began early in the syngnathid lineage. It is conceivable that changes in the amino acid sequence of syngnathid Pitx1 have had functional consequences for the protein’s interaction with its gene targets (such as *tbx4*), affecting hindlimb development. We found no pipefish ortholog of *tbx4*. Failure to find pipefish *tbx4* in the genome assembly does not necessarily mean the gene has been evolutionarily lost; however, the possible loss of this gene with an apparently narrow developmental role in teleosts – in hindlimb development [46] – is consistent with the evolutionary loss of the hindlimb itself in syngnathids. Loss of the pelvic fins in a syngnathid ancestor may have occurred shortly before or after the origin of the lineage, because the closest extant relatives – the ghost pipefishes (Family Solenostomidae) [72, 73] – have large, clasping pelvic fins in which females brood the embryos [74]. Interestingly, Pitx1 in robust ghost pipefish (*Solenostomus cyanopterus*) lacks the homopolymeric repeats described above (Fig. 6).

A family of homeodomain transcription factors important for limb, brain, and craniofacial development, the Dlx genes, are arranged in gene pairs associated with specific Hox clusters. Within and near the Dlx gene pairs are CNEs recognizable by alignment among sequences from even distantly related vertebrates. Several teleost *dlx* clusters, for example, have CNEs in common with mammals [40, 75]. Putatively these CNEs are preserved because they have a function, perhaps in regulating gene expression of the *dlx* genes themselves. For instance, two CNEs that fall between *dlx1* and *dlx2* and that are conserved between teleosts and mammals direct reporter gene expression in the developing forebrain and first and second pharyngeal arches in murine [43] and in zebrafish [75] embryos. We found that pipefish has retained these two ancient CNEs but has apparently lost a third element that is as deeply conserved (i.e. between mammals and teleosts), from within an intron of *dlx1a*. In addition, at least two more CNEs in the intergenic region of *dlx1/2a* that are conserved among other percomorphs are lost or diverged beyond recognition in pipefish (Fig. 5). Experimental mutation of mouse *Dlx1/2* genes creates defects in the development of pharyngeal arch derivatives, such as the mandible and teeth [76]. Knockdown of these genes in zebrafish causes embryos with shortened faces and mispatterning of first and second arch cartilages and a reduced ethmoid (a cartilage of the ventral neurocranium) [77]. In addition, *dlx2* genes are expressed in developing teeth in cichlids, catfish, and cyprinids [78–80], and *dlx2a* is expressed in migrating neural crest that will form the anterior pharyngeal arch cartilages [77, 81]. Pipefish embryos show modified development of the anterior skull including cartilage derivatives of the first and second pharyngeal arches, particularly elongation of the hyosymplectic (a cartilage of the second arch), as well as unusual early curvature and later elongation of the ethmoid cartilage (see Additional file 1: Figure S7 for a view of pipefish craniofacial development), implicating changes in expression of early acting genes such as *dlx2a*, involved in cranial neural crest survival and patterning. Functional testing in other teleosts could reveal whether the CNEs here shown to be erased in pipefish are functional units that modulate expression of the *dlx1/2a* cluster genes and possibly affect pharyngeal arch or tooth development.

Male pregnancy in syngnathid fishes is a true example of evolutionary novelty. In many lineages, including *S. scovelli*, males gestate developing embryos in a tightly regulated environment defined by a complex brood pouch. Extensive cellular and developmental changes in the pouch occur leading up to and during pregnancy, including proliferation of epithelial cells, development of specialized secretory cells, and angiogenesis [10, 82, 83]. These specializations are likely the consequence of adaptation, as they enable functions directly relevant to fitness, including solute, gas, and nutrient delivery to a male’s brood [12, 13, 84], as well as immune priming of offspring [85]. Consistent with this functional diversity, our genome-based analysis of male pregnancy in *S. scovelli* revealed a transcriptionally rich brood pouch in which over 73% of annotated genes were expressed robustly and over 1000 were differentially expressed as a consequence of pregnancy (Additional file 2: SH2). Previous studies, based on de novo transcriptome assemblies, characterized pregnancy-specific gene expression in pipefish species of *Syngnathus* [52] and in the seahorse *Hippocampus abdominalis* [54], but lack of a reference genome in those surveys limited insights into the transcriptional breadth of the pouch and single gene resolution for transcript abundance measurements. Our differential expression analysis comparing early-stage pregnant to non-pregnant male pouch tissue echoes many of the patterns described in the comprehensive seahorse study [54], including evidence for positive regulation of developmental processes, lipid transport, homeostasis, and the immune system during pregnancy. Interestingly, we noted a more pronounced signature of pregnancy-specific gene expression for innate, relative to adaptive, immune pathways in Gulf pipefish (Additional file 1: Figure S6). This observation is likely in part a consequence of pipefishes in *Syngnathus* having lost important genetic components of MHC class II mediated immunity [51], although MHC class I components remain intact. Syngnathid fathers face unique demands with respect to immunity and pregnancy, given that the brood pouch is a non-urogenital organ more directly exposed to the environment than internal uterine structures of other vertebrates. A seemingly difficult balance among pathogen control, maintenance of beneficial microbes, and mitigation of attack against non-self (embryonic) tissues must therefore be struck. Although future work regarding the details of this balance will be required to say so, perhaps a uniquely fine-tuned division of labor between innate and adaptive immunity has been an evolutionary outcome of male pregnancy, a balance we hypothesize differs across syngnathid lineages with varying brood pouch complexity.

The significance of gene duplication to adaptation and biological diversification in general is continually of interest to evolutionary biologists [86–88]. We identified at least four clustered members of the *patristacin* gene subfamily on a single scaffold of LG4 in the Gulf pipefish genome (Fig. 7). Given the striking patterns of gene expression for *pastn1* and *pastn2* with respect to pregnancy, it is possible that gene duplication followed by neo- or subfunctionalization played a key role in the evolution of male pregnancy, although surveys of other syngnathid genomes and those of their closest relatives are needed to test this hypothesis. Our interpretation of the evolution of *patristacins* is distinct from that of Harlin-Cognato et al. [18], who suggested that one *patristacin*, identified without the advantage of a complete *S. scovelli* genome, took on a novel role in male pregnancy by a spatiotemporal shift in gene expression and not via gene duplication. Our genome-wide approach has provided additional information, however, by revealing the complete coding sequence for multiple *patristacin* paralogs in *S. scovelli*. Because the two *patristacins* with exceptional pregnancy-specific gene expression (*pastn1* and *pastn2*) likely diverged by gene duplication after pipefish separated from the other fish lineages in our comparison, we provide evidence for a role of relatively recent gene duplication in *patristacin* evolution. Our phylogenetic analysis highlights a second, large expansion of *patristacin*-like genes in the genome of *Xiphophorus maculatus*, suggestive of high duplicate retention in multiple live-bearing fish lineages.

The specific functional roles *patristacins* play in male pregnancy are currently unknown, but our current phylogenetic understanding of their place among teleost Astacin-like metalloproteases suggests that they may be more functionally similar to Nephrosin-like proteins than hatching enzyme components (Fig. 7a, Additional file 1: Figure S8). Kawaguchi et al. [89] showed, for example, that medaka 6-cysteine astacin genes *mc6ast1* and *mc6ast2*, orthologs of zebrafish *c6ast1* and zebrafish *c6ast3/4*, respectively, were expressed in a wide range of tissues, in contrast to medaka hatching enzymes, which were expressed exclusively in pre-hatching embryos. Another member of this gene subclade, *cimp1*, is expressed epithelially in the developing cichlid jaw and may play a role in ECM turnover during development [90]. We hypothesize that *patristacins* evolved from an already transcriptionally promiscuous ancestor and now, following subsequent duplication events, work in concert to regulate the remodeling of the pouch epithelium necessary for the sustenance of pregnancy. Our characterization here of their structural organization and expression patterns in the brood pouch will inform and facilitate future functional studies of these gene duplicates and their specific roles in male pregnancy.

## Conclusions

We present the first annotated reference genome assembly, organized into chromosomes, for a syngnathid fish. Our comparisons of the Gulf pipefish genome to other fish genomes reveal two chromosomal fusions in the syngnathid lineage. We provide additional evidence suggesting that syngnathiform fishes are an outgroup relative to fellow percomorph fishes commonly used in comparative genomics studies. The Gulf pipefish genome will therefore serve as a useful comparator in studies that aim to understand rates of genome evolution among percomorphs for which there are existing genomic resources. We show that losses of both genes and CNEs have occurred in pipefish gene families important for vertebrate craniofacial, tooth, hindlimb, and axial development, all features that are highly modified in syngnathids. In addition, we detail aspects of the molecular biology of male pregnancy, a unique and unifying feature of the pipefish, seahorses, and seadragons; in particular, we exploited the annotated Gulf pipefish genome and transcriptional profiling to show how pregnancy is associated with clear changes in gene expression in the male brood pouch tissue, a broad example being regulation of the innate immune system and a specific example being regulation of duplicated *patristacins*.

## Methods

### Genome sequencing libraries and genome sequence assembly

We isolated genomic DNA from a single adult male pipefish purchased from Gulf Specimen Marine Laboratories, Inc. (Panacea, FL, USA) in 2010 using standard organic extraction. We generated four different 100 nt paired-end Illumina libraries for whole genome shotgun assembly: (1) a short (~180 bp) insert length library; (2) a 2.5–5 kb insert length jumping library; (3) a 5–10 kb insert length jumping library; and (4) a 11–15 kb insert length jumping library. To construct the 180 bp library, we sheared 1 μg of genomic DNA to less than 500 bp using sonication in a Bioruptor (Diagenode) and size selected fragments by agarose gel electrophoresis, followed by end repair of the fragments, addition of adenosine overhangs, ligation of Illumina sequencing adapters, and 12 cycles of PCR amplification with Phusion polymerase (NEB). We used the Illumina Nextera Matepair Sample Preparation Kit (Illumina, cat. #FC-132-1001) to generate the three jumping libraries. Briefly, we performed a single tagmentation reaction using 5 ng of genomic DNA, selected the three aforementioned fragment size ranges using agarose gel electrophoresis, and performed the remaining library preparation steps in parallel, including circularization, shearing by Bioruptor (30 s on, 60 s off, for 15 min), streptavidin bead pull-down, end repair, addition of adenosine overhangs, Illumina indexed adapter ligation, and 15 cycles of PCR amplification. We sequenced the short-insert library (two lanes) and three jumping libraries (all in one lane) on an Illumina HiSeq2000 at the University of Oregon Genomics Core Facility (UOGCF).

To minimize the inclusion of sequencing adaptors, sequencing errors, and repetitive DNA sequences in the assembly process, we used tools from the Stacks software suite [91, 92] to adaptor-trim and discard low-quality read pairs (*process_shortreads*) and filter pairs containing abundant k-mers (*kmer_filter*). Remaining were 238.6 million overlap pairs, 3.5 million 11–15 kb mate-pairs, 21.6 M 5–10 kb mate-pairs, and 44.4 M 2.5–5 kb mate-pairs, which we used for assembly with ALLPATHS-LG [21]. Because initial k-mer spectrum analyses suggested a highly polymorphic genome, we ran ALLPATHS-LG with HAPLOIDIFY = TRUE. To assess completeness of the assembly with respect to CEGs, we used CEGMA [22]. For a summary of all Illumina sequencing data used in the assembly, see Additional file 3.

We confirmed several apparent pipefish gene losses via comparison among preliminary genome assemblies derived from independently constructed molecular libraries and generated using SGA [93] and Velvet [94] and via targeted Sanger sequencing. Briefly, SGA and Velvet assemblies incorporated a shotgun genomic DNA library with an insert length of 470 nt, sequenced independently with 120 nt, 100 nt, and 80 nt paired-end Illumina reads. For the SGA assembly, the overlap value was optimized to 70 during the contig construction phase. Scaffolding was performed using SSPACE [95], with the three mate-pair libraries mentioned above and an additional 2–8 kb mate-pair library. These analyses filled seven small gaps in the range of 51–1753 nt in the *hoxba*, *hoxbb*, *hoxca*, and *hoxda* clusters. The degraded nature of *hoxa7a* was also confirmed by Sanger sequencing.

### RNA-seq libraries and transcriptome assemblies

#### Embryo and fry transcriptome

Embryos, flushed from the pouch of lab-reared pregnant males, and fry were euthanized in Tricaine-S and stored in RNA-Later (Ambion). Tissue including the head to just posterior to the pectoral fin was dissected and pooled from 17 embryos (including 15 at 8 days post fertilization (dpf) and 2 at 10 dpf) and from 18 fry (including 2 at 16 dpf and 16 at 17 dpf). Double-stranded complementary DNA (cDNA) was produced from these tissues via standard methods including RiboPure Kit (Ambion) for total RNA isolation, MicroPoly(A)Purist Kit (Ambion) for messenger RNA (mRNA) enrichment, mostly hexameric Random Primers (ThermoFisher, #48190-011) and Superscript III reverse transcriptase (Invitrogen) for first strand synthesis, and Random Primers with Kleno exo-DNA polymerase (Epicentre). Paired-end Illumina sequencing libraries were created using standard methods including mechanical shearing of the cDNA and TA ligation of adaptors (top, 5′ACACTCTTTCCCTACACGACGCTCTTCCGATC*T3′; bottom, 5′Phos-GATCGGAAGAGCGGTTCAGCAGGAATGCCGAG3′), slab gel size fractionation to isolate fragments in the 200–500 bp range, and amplification using Illumina-compatible primers (5′AATGATACGGCGACCACCGAGATCTACACTCTTTCCCTACACGACGCTCTTCCGATCT3′ and P2 reverse primer, 5′CAAGCAGAAGACGGCATACGAGATCGGTCTCGGCATTCCTGCTGAACCGCTCTTCCGATCT3′). The library was sequenced on an Illumina GAIIx platform to produce 60 nt paired-end reads and on an Illumina HiSeq2000 platform to produce 100 nt paired-end reads (see Additional file 3 for details).

#### Male brood pouch

Six non-pregnant and six early-stage pregnant adult males were captured from Redfish Bay, TX, USA (Lat: 27.86795057508745, Long: –97.08869218576297), transported to the laboratory, and euthanized as described above approximately 24 h after capture. We carefully dissected all brooding tissues, including the pouch “flaps” and epithelium, but excluding all embryonic tissue in the case of pregnant males. We fixed tissues in RNA-Later (Ambion) before freezing, homogenized by pestle upon thawing, and isolated total RNA using Trizol Reagent (Invitrogen) and RNeasy MinElute columns (Qiagen). A unique RNA-seq library was generated for each individual from 1 ug of total RNA using the TruSeq RNA v2 Kit (Illumina) and the 12 mRNA-seq libraries were sequenced across two lanes of Ilumina HiSeq 2000, generating 100 nt paired-end reads.

#### De novo transcriptome assemblies

We removed low-quality and adaptor sequences from RNA-seq reads using *process_shortreads* from Stacks [91, 92], overlapped paired-end reads using FLASH [96], and performed rare k-mer filtering and digital normalization using *kmer_filter* from Stacks. We then generated two separate de novo transcriptome assemblies (one for each tissue type) from the cleaned, filtered RNA-seq data using Trinity [97] with --*min_kmer_cov* set to 3.

### Genome annotation

Prior to genome annotation, the assembly was soft-masked for repetitive elements and areas of low complexity with RepeatMasker [98] using a custom Gulf pipefish library created by RepeatModeler [99], Repbase repeat libraries [100], and a list of known transposable elements provided by MAKER [25]. In total 15.36% of the genome assembly was masked by RepeatMasker. Repetitive elements were annotated with RepeatModeler. Hidden Markov models (HMMs) for gene prediction were generated by SNAP [101] and Augustus [102] and were iteratively trained for the assembly using MAKER as described by Cantarel et al. [103]. Training was performed on the five largest scaffolds and two additional scaffolds that were UTR rich, totaling 25 Mb. Evidence used by MAKER for annotation included Gulf pipefish mRNA-seq transcriptomes from embryonic head tissue and brood pouch tissue (assembled with Trinity – see above), protein sequences from threespine stickleback (*Gasterosteus aculeatus*), zebrafish (*Danio rerio*), medaka (*Oryzias latipes*), and tilapia (*Oreochromis niloticus*) (downloaded from Ensembl: Broad S1, GRCz10, HdrR, Orenil1.0, respectively), and all Uniprot/swissprot proteins [104].

We filtered the annotations by MAKER to include evidence-based annotations with assembled transcriptome or protein support and those ab initio gene predictions that contained protein family domains as detected with InterProScan [105]. Gene annotations were manually refined for *hox*, astacin-like metalloprotease, and *pitx* genes. For each annotated amino acid sequence we queried the NCBI nr database using BLASTP and compiled the results for the top BLASTP hit per gene in Additional file 2: SH6.

### Linkage map and map integration

#### Mapping cross

For the genetic cross, wild male and female *S. scovelli* were captured from Redfish Bay and maintained in the lab. A total of six sequential broods from a single mated pair, totaling 108 F1 progeny, including fry from the brood pouch plus 15 collected just prior to emergence, were gathered and flash frozen over a span of 4 months. Genomic DNA was isolated from individual progeny and from their parents via the Qiagen DNeasy Kit. RAD-seq libraries were made using the restriction enzyme SbfI as in Baird et al. [106], Hohenlohe et al. [107], and Etter et al. [108] with the Illumina-compatible, barcoded P1 adapters and primer types used in Hohenlohe et al. [109] and the P2 adapter type used in Hohenlohe et al. [107]. Single-end reads of 100 nt were produced from two lanes on an Illumina HiSeq2000 (see Additional file 3 for details). The parents were sequenced to greater depth than progeny (see below) to make an accurate catalog of diploid genotypes possible in the cross.

#### Marker genotyping

The two lanes of Illumina data resulted in 367,085,475 raw reads which were analyzed using the software, Stacks [91, 92]. Using the *process_radtags* program, reads were demultiplexed according to barcode and discarded if the barcode could not be determined after correcting for sequencing error, if the restriction enzyme cut site was not intact, or if the sequencing quality was too degraded. The 218,309,324 remaining reads were analyzed by the Stacks de novo pipeline to assemble and genotype the RAD loci. A minimum of three identical reads (–m 3) was required to form a “stack” or putative allele in each individual, up to five differences were allowed when merging stacks into putative loci (–M 5) and up to 3 differences were allowed when merging loci from different individuals into the catalog (–n 3) to accommodate fixed differences between the cross parents. The *genotypes* program from Stacks was used to export data in a CP cross-format for use in JoinMap and the genotypes were uploaded to the Stacks web interface. Genotype data with markers present in at least 75 of the 108 individual progeny were exported from the web interface for linkage analysis.

#### Map construction

Linkage analysis was performed with JoinMap 4.1 [110] using only markers that were present in at least 75 of the 108 individual progeny. Markers were initially grouped in JoinMap 4.1 using the “independence LOD” parameter under “population grouping” at a minimum LOD value of 15.0, and markers that remained unlinked at LOD < 15 were excluded. Marker sets were partitioned into paternal and maternal markers to enable the construction of sex-specific linkage maps. Marker ordering was performed using the Maximum Likelihood (ML) algorithm in JoinMap 4.1 with default parameters. Supposed double recombinants were identified using the “genotype probabilities” feature in JoinMap 4.1 and by visual inspection of the colorized graphical genotypes in the male, female, and consensus maps. After visual inspection of the individual sequences in the web interface of Stacks, markers were manually corrected as needed in the web interface and re-exported. For example, if a double recombinant was a homozygote with a small number of sequences, the genotype was eliminated because it might represent a heterozygote with no sequences for the second allele. Conversely, if the double recombinant was a heterozygote with only one sequence for the second allele, the genotype was eliminated because the second sequence could be sequencing error. The new dataset with corrected genotypes was loaded again into JoinMap 4.1 and the process was repeated until no suspect genotypes were identified. The “expected recombination count” feature in JoinMap 4.1 was used to identify individuals with higher than expected recombination events; marker order was visually inspected and, when necessary, optimized by moving a marker or sets of markers to a new map position that reduced the number of recombination events. When a marker or sets of markers could be in multiple map positions, the markers were moved to a position congruent with their physically aligned scaffold location if there was no cost to the map.

#### Integrating the assembly and the linkage map

The 4375 markers from the linkage analysis were integrated with the assembled pipefish scaffolds to create a chromonome using the software, Chromonomer (http://catchenlab.life.illinois.edu/chromonomer/). Markers were aligned to the set of assembled pipefish scaffolds using GSnap [111], requiring unique alignments, allowing up to five mismatches (–m 5), counting gaps as four mismatches (–i 4), and requiring 99% of the RAD locus to align (--min-coverage = 0.99). The AGP file produced by ALLPATHS-LG that describes the assembly, the linkage group, and map position of the markers in the map, the alignments of the markers to the scaffolds, and the FASTA file containing the sequence from the assembly are all fed into Chromonomer, which integrates them in the following way. First, markers are arrayed along the scaffolds they are aligned to and scaffolds that have markers from more than one linkage group are identified (no scaffolds were split between linkage groups). A coherent ordering of markers must be found for each scaffold so that physical basepair and map position are consistent among all markers for that scaffold. Markers that are out of order with respect to the map or scaffold are discarded (unless it is the last marker holding a scaffold into the map). Of the 4375 markers, 649 were excluded in this phase, leaving 3726 markers in the final “chromonome.” If a scaffold spans more than one map position, and physical order is the same as map order, the orientation of the scaffold is positive. If physical and map order are inverted, the scaffold is considered in negative orientation and the sequence is reverse complemented. Otherwise orientation is unknown and the scaffold remains in positive orientation by default. Scaffolds are then hung from the linkage group they occur on, according to map position. Ordered markers may place the scaffold in more than one place within the linkage group, that is, one or more scaffolds occur within the focal scaffold according to the linkage map. This can be due to an incorrect assembly join or because a smaller scaffold is filling a gap in a larger scaffold. In these cases, the scaffold is split at the largest gap that can be found between the markers in the map that indicate where the split must occur. Starting with 553 scaffolds, five scaffolds were split one time each for a total of 558 scaffolds in the chromonome. Sequence from the scaffolds is then concatenated into chromosomes according to the orientation and integrated order with standard 100 bp gaps placed in between each join resulting in a chromonome of 266,330,253 bp (53.6Kb scaffold join gaps) with 40,734,039 bp of sequence remaining in unintegrated scaffolds. Finally, the genome annotation is translated to the new chromonome providing a genome-level ordering of genes for use in conserved synteny analysis and new AGP, FASTA, and GFF files are generated to describe the chromonome.

### Conserved synteny analysis

In order to visualize evolutionarily conserved gene neighborhoods, i.e. conserved synteny, we used the Synolog software (Catchen, unpublished). We used Synolog to identify orthologs between the Gulf pipefish, threespine stickleback, medaka, green spotted pufferfish (*Tetraodon nigroviridis*), zebrafish*,* spotted gar, and southern platyfish and to identify conserved gene neighborhoods pairwise between the different species. Genome-wide images of conserved synteny were drawn by Synolog by combining the conserved synteny blocks across the genome and incorporating the integrated linkage map/assembly output by Chromonomer where appropriate (Fig. 2c). Protein gene models for each non-pipefish species were downloaded from Ensembl. While Synolog is a new and independent implementation, the algorithm to identify conserved synteny and the biological inferences stemming from its application are as described in Catchen, et al. [112].

### Phylogenomic analysis using ultraconserved elements

We added UCEs from Gulf pipefish, Pacific bluefin tuna, and southern platyfish genomes to an existing UCE dataset containing sequences for 27 actinopterygiian fishes and published by Faircloth et al. [32]. To retrieve each of the 491 UCEs from the three genomes above, we generated a consensus sequence of each alignment from Faircloth et al. [32] using *em_cons* from EMBOSS [113], searched for each consensus sequence in each genome using LASTZ [114], and extracted unique search hits from each genome using BEDTools [115]. For this we used the tuna reference genome available from http://nrifs.fra.affrc.go.jp/ResearchCenter/5_AG/genomes/Tuna_DNAmicroarray/index.html and the platyfish genome from Ensembl. We obtained 457, 453, and 479 single-copy UCEs for Gulf pipefish, tuna, and platyfish, respectively. A multiple sequence alignment for each UCE was generated using MAFFT v7 [116] with options --localpair and --maxiterate 1000, and minor manual adjustments were made when necessary.

We performed substitution model selection for each UCE alignment using the corrected Akaike Information Criterion, as implemented in jModeltest-2.1.10 [117, 118]. The GTR + gamma model was selected for the largest percentage of the total aligned sequence data. We concatenated UCE alignments, ordering them so that the loci having the same best-fitting substitution model were grouped together. We proceeded with a partitioned phylogenetic analysis using the concatenated alignment (153,032 nt total), and the GTR + gamma model for all partitions. Maximum likelihood (ML) phylogenetic inferences were conducted with RAxML version 8.2.4 [119] using default settings. We produced a consensus ML tree using the rapid bootstrap search algorithm described in Stamatakis et al. [120]. Briefly, 1000 rapid bootstrap searches were conducted, followed by fast ML searches on 200 of these, followed by a slow ML search on the 10 best fast ML trees. Clade confidence was assessed with SH-aLRT support values and bootstrap replicate frequencies. We specified *Polypterus senegalus* as the outgroup for tree rooting.

### Characterization of *hox* clusters

#### *hox* gene content

Teleost *hox* gene sequences acquired from Ensembl were used as queries for BLAST searches of the final Gulf pipefish genome assembly using Geneious (version 8.0.5). Exon boundaries were annotated by hand using alignments with the query *hox* genes. The *hox* genes annotated in the Gulf pipefish assembly were then BLAST-searched against the NCBI NR sequence database to confirm gene identity using Geneious (version 8.0.5). Additionally, *hox* genes were identified, following the method outlined above, in the Pacific bluefin tuna genome (see genome source above) [121].

*hox* cluster microRNAs and long non-coding RNAs within the *hox* cluster were identified using VISTA analyses based on CNEs within *hox* clusters across Gulf pipefish, threespine stickleback, mouse (*Mus musculus*), spotted gar, zebrafish, Pacific bluefin tuna, medaka, and fugu (*Takifugu rubripes*) [41, 42, 122–124]. We aligned primary miRBase [125] microRNA sequences from stickleback, zebrafish, medaka, and fugu to *S. scovelli hox* regions using MUSCLE [126] to supplement annotations. The hairpin loops of the annotated microRNAs were confirmed using RNAfold (http://rna.tbi.univie.ac.at/cgi-bin/RNAWebSuite/RNAfold.cgi). When known *hox* cluster microRNAs were not detected in the Gulf pipefish genome, we further confirmed absence of the conserved seed sequence, which was the case for *mir196b* between *hoxb13a* and *hoxb9a* and *mir10a* between *hoxb5b* and *hoxb3b*. All conserved non-coding sequences annotated within the Gulf pipefish hox cluster were queried against miRBase Sequence Databases (Release 21) for mature miRNA chordate sequences and miRNA chordate hairpins (downloaded from miRBase) using BBMapSkimmer [127] for further identification of microRNAs. Kmer index size was set to 7, max indel set to 0, approximate minimum alignment identity set to 0.50, secondary site score ratio set to 0.25, behavior on ambiguously-mapped reads set to retain all top-scoring sites, and maximum number of total alignments to print per read set to 4 million. See Additional file 2: SH7 for scaffold locations and sequences of microRNAs and long non-coding genes.

### Characterization of *dlx* CNEs

CNEs between *dlx1* and *dlx2*, between *dlx3* and *dlx4*, and between *dlx5* and *dlx6* were identified using mVISTA analyses based on levels of sequence conservation within *dlx* clusters across Gulf pipefish, Atlantic cod, threespine stickleback, zebrafish, human, Pacific bluefin tuna, medaka, and fugu [41, 42, 122–124]. Sequences were downloaded from Ensembl for cod, stickleback, zebrafish, human, medaka, and fugu. Tuna sequences were downloaded from the reference genome source cited above. Medaka was set as the reference sequence for the *dlx1/2* and *dlx5/6* comparisons and stickleback was the reference for the *dlx3/4* comparisons. Alignment of each sequence from these species were aligned using the shuffle-LAGAN algorithm through the mVISTA website under default parameters. See Additional file 2: SH7 for scaffold locations of CNEs.

### Characterization of pelvic fin development candidates

Pitx1, Pitx2, and Pitx3 protein sequences were obtained from our pipefish annotation, Ensembl, and Genbank (in the case of stickleback Pitx1) for human, coelacanth (*Latimeria chalumnae*), spotted gar, zebrafish, blind cavefish (*Astyanax mexicanus*), medaka, tilapia, green spotted pufferfish, and threespine stickleback, and aligned using MAFFT (with default settings). To isolate DNA fragments for Sanger sequencing of *pitx1* from the messmate pipefish (*Corythoichthys haematopterus*) and the robust ghost pipefish (*Solenostomus cyanopterus*) genomic DNA, we designed degenerate PCR primers (in IUPAC notation, forward 5′-CGGAGCGCAACCAGCARATGGA-3′ and reverse 5′-GGACGACGACATGSCSCWGTTGAT-3′) for amplification using Phusion DNA polymerase (New England Biolabs) in Phusion HF buffer, and an annealing temperature of 55 °C.

Because *tbx4* was not represented in the pipefish genome annotation, we attempted to determine its location in the genome assembly manually by using a targeted profile HMM generated from several aligned teleost Tbx4 protein sequences. HMM-based approaches are more sensitive than BLAST-based approaches when searching for divergent homologs [128], a possible scenario when a gene has evolved rapidly or has degenerated. Briefly, we used an alignment of Ensembl Tbx4 sequences from spotted gar, zebrafish, medaka, southern platyfish, threespine stickleback, green spotted pufferfish, and tilapia to generate a profile HMM with hmmer2 [129], then searched for sequences in the Gulf pipefish genome with this model using the genewisedb program of wise2 (http://www.ebi.ac.uk/~birney/wise2/) with default search settings.

### Differential expression analysis

We aligned adaptor- and low-quality-trimmed, forward reads from the 12 brood pouch RNA-seq libraries to the annotated Gulf pipefish genome using GSnap [111]. We counted the number of uniquely mapped reads per exonic region of each annotated gene using HTSeq-count [130] and used the counts to test for differential gene expression between pregnant and non-pregnant males using the negative binomial exact test [131], after TMM normalization, implemented by the R/Bioconductor package edgeR [132]. We limited differential expression analysis to those genes with at least one read per million counted (cpm) in at least four of the 12 fish, which reduced the dataset to 15,253 genes.

To connect genes annotated in the pipefish genome with putative functional information, we mapped the pipefish amino acid sequences to KEGG Orthology (KO) entries [133] using the KEGG Automatic Annotation Server [134]. We then identified KEGG PATHWAYS enriched for pipefish KOs with extreme log_2_ fold change values from the pregnancy differential expression analysis using the R/Bioconductor package GAGE [135]. To visualize individual members of KEGG PATHWAYS enriched for pregnancy-sensitive genes we used the R/Bioconductor package Pathview [136]. We also used Ensembl IDs for putative *D. rerio* orthologs of Gulf pipefish genes to test for overrepresentation of PANTHER GO-slim Biological Process terms among pregnancy-enriched and pregnancy-depressed genes using binomial tests implemented by the online resource PANTHER (pantherdb.org), [137, 138]. For the overrepresentation tests, we used all genes tested for differential expression (see above) and matched with a zebrafish ortholog as the comparison set. To interpret the results of overrepresentation tests for pregnancy-enriched and pregnancy-depressed sets we only considered GO-Slim terms represented in the comparison set by at least five genes and we controlled the FDR at 0.1 as in Benjamini and Hochberg [139]. Results for these overrepresentation tests are in Additional file 2: SH4 and Additional file 2: SH5.

To visualize and quantify multivariate differences among individual brooding tissue samples in transcript space, we calculated Bray-Curtis dissimilarity based on TMM-normalized cpm values, performed non-metric multidimensional scaling (nMDS), and conducted permutation-based multivariate analysis of variance (perMANOVA) to test for a global transcriptional effect of pregnancy status, all using the R package vegan [140]. Similarly, to visualize clustering of genes and pouch libraries via co-expression patterns, we generated heatmaps for all pouch-expressed genes and several immune system related KEGG pathways. Ward clustering was used, based on Euclidean distance calculated from scaled, log_2_-transformed cpm values, implemented by the R function hclust. Unless noted otherwise, all additional analyses related to the gene expression were conducted using core packages within the statistical programming language R [141].

### Characterization of *patristacins*

Previous work identified members of the astacin-like metalloprotease gene family as candidates for playing a functional role in male pregnancy [18, 52]. We confirmed extreme transcriptional differences for two of these *patristacins* between brood pouch tissue of pregnant and non-pregnant males (see “Differential expression analysis” section) and set out to characterize the distribution of this gene family in the Gulf pipefish and other teleost genomes. We compared protein sequences from pipefish gene annotations bearing similarity to *patristacins* against the Ensembl zebrafish GRCz10 protein set using BLAST and discovered that all similar zebrafish homologs belong to Ensembl protein family ENSFM00500000270265 (choriolytic enzymes). We used all actinopteryigiian fish sequences from this Ensembl protein family alignment to generate a HMM profile using hmmer2 [129], then searched for similar sequences in the Gulf pipefish genome using the genewisedb program of wise2 (http://www.ebi.ac.uk/~birney/wise2/) with default search settings. These protein family-specific annotations allowed us to both correct and supplement initial MAKER annotations as necessary. Most of the *S. scovelli* astacin-like metalloproteases annotated in this manner, including at least four tandemly arrayed patristacins on scaffold 62, shared high sequence similarity with zebrafish homologs from Ensembl protein family ENSFM00500000270265. Six of the *S. scovelli* astacin-like metalloproteases were most similar to three additional Ensembl protein families, including ENSFM00500000282854 (Metalloendopeptidases), ENSFM00570000851071 (Bone morphogenetic 1/Tolloid-like proteins), and ENSFM00500000270104 (Meprins).

To identify potential *patristacin* orthologs and/or close paralogs in several teleost genomes, we repeated the HMM search using a hmmer2 profile generated from an alignment of the four pipefish *patristacins*, but included the Gulf pipefish assembly, and the Ensembl genomes of spotted gar, zebrafish, platyfish, and green spotted pufferfish as targets. Hits from these searches were used to understand the evolution of patristacins in the syngnathid lineage. Excluding hits that corresponded to the more distantly paralogous Bmp1/Tolloid-like and Merprin proteins [142], with the exception of Meprin1b as an outgroup (see Fig. 7), we aligned all unique astacin-like amino acid sequences from the aforementioned actinopterygii genomes with MAFFT v7 [116] using options --localpair and --maxiterate 1000. We then made manual adjustments to the alignment by removing non-conserved residues at the ends, yielding a final alignment of 55 sequences, covering 269 amino acids. We used the PhyML 3.0 web server [143] for Akaike Information Criterion model selection and ML phylogenetic inference. The WAG + G + I + F model was selected and we proceeded with two separate evaluations of ML tree clade support: PhyML’s fast SH-like aLRT and 500 bootstrap replicates.

## Additional files

Additional file 1: This pdf file contains the following supplementary figures: S1–S8. Legends for these figures are presented at the beginning of Additional file 1. (PDF 3.97 mb)
Additional file 2: This xlsx file contains the following supplementary spreadsheets, each included as a separate tab in a single Microsoft Excel File: SH1–SH7. Descriptions for these spreadsheets are presented at the beginning of Additional file 2. (XLSX 2.47 mb)
Additional file 3: This doc file is a comprehensive summary of Illumina data used for the Gulf pipefish genome project. A diverse collection of short-read sequencing data was used to understand the genome of *S. scovelli*. Included are descriptions of library types, raw read counts, and analyses in which the different libraries were used. (DOCX 90 kb)
