# Supplementary material for: The genome of the Gulf pipefish enables understanding of evolutionary innovations
Source: Genome Biol. 2016 Dec 20;17:258. doi: 10.1186/s13059-016-1126-6 (PMC5168715; doi:10.1186/s13059-016-1126-6)
Supplement: Additional file 1: — This pdf file contains the following supplementary figures: S1–S8. Legends for these figures are presented at the beginning of Additional file 1. (PDF 3.97 mb) [file 13059_2016_1126_MOESM1_ESM.pdf]

Additional File 1 (Supplementary Figures)
This .pdf file contains the following supplementary figures: S1-S8. Legends for these figures are below.

S1 The *S. scovelli* genetic maps. a) The consensus map, which combines markers from both maps for each linkage group (LG), was used to link the scaffolds into chromosomes. b) Regional patterns of reduced recombination in males (inferred to be centromeric) and reduced recombination in females (inferred to be telomeric) were used to orient of centromeres to the top in the LGs in panel (a). Blue marks regions of relative expansion in the female map; red marks relative expansion in the male map for the markers they have in common on this example linkage group. c) On LG 1 a large block of markers (shown in red) aligning to about 17 Mb of scaffold length is non-recombining in both the male and female in this genetic cross. A possible explanation for this is that both parents carry a large inversion across this region. This is one of the two chromosomes formed by fusion of two ancestral chromosomes.

S2 Length distributions for unlinked scaffolds and for those captured by linkage groups show that the majority of large, gene-containing scaffolds are incorporated into the chromosome models. a) Boxplots and individual points reflect scaffold length distributions. Individual points (scaffolds) are coded by gene density (grading from yellow for no genes to red for high gene density). b) Scaffold length distributions in histogram form.

S3 VISTA plots suggest pipefish *dlx* clusters are missing CNEs (red arrows) that are conserved between other percomorphs and cod, a non-percomorph. The reference for the *dlx3/4a* and *3/4b* plots is stickleback (Gac) and for *dlx5/6a* is medaka. Hsa, human; Dre, zebrafish; Gmo, cod; Ssc, pipefish; Ola, medaka; Tru, pufferfish. Exons are highlighted in blue, CNEs in pink. Zebrafish has evolutionarily lost *dlx3a*. See Fig. 3 for the *dlx1/2a* VISTA comparison.

S4 Gene co-expression patterns reveal brood pouch transcriptome differences between pregnant and non-pregnant males. a) nMDS ordination of samples highlighting differences in reduced (2)-dimensional space. Orange circles represent pregnant male fish and blue boxes non-pregnant fish. b) Heatmap including all 15,253 pouch-expressed genes with sample (column) and gene (row) clustering. c) Heatmap including 70 randomly selected pouch-expressed genes with KEGG orthology (KO) assignments. d) Heatmap including the 70 pouch-expressed genes with “natural killer cell mediated cytotoxicity” KO assignments. Pink boxes highlight KO descriptions for clusters of genes showing pregnancy-depressed (above) and pregnancy-enriched (below) expression. Color keys for all heatmaps show expression values, scaled for each gene to have a mean of 0 and a standard deviation of 1, and dashed portions of clustering dendrograms represent non-pregnant males.

S5 Pathview [132] KEGG pathway renderings with scaled pregnancy-specific log_2 fold changes mapped to KOs. Blue hues represent pregnancy-depressed KOs, while orange hues represent pregnancy-enriched KOs. a) The "coagulation and complement cascades" KEGG pathway. The red asterisk marks pregnancy-enriched components of the Membrane Attack Complex (see text). b) The "cytokine-cytokine receptor interaction" KEGG map, with red asterisk marking the chemokines, of which pro-inflammatory chemokines are pregnancy-enriched.

S6 Heatmaps comparing pregnancy-specific expression patterns for an innate immunity KEGG pathway [a), "coagulation and complement cascades"] and an adaptive immunity KEGG pathway [b), "antigen processing and presentation"]. The pink box highlights pipefish genes in the coagulation and complement cascades that are distinctly pregnancy enriched. No such strong patterns are observed for antigen processing and presentation. Color keys for heatmaps show expression values, scaled for each gene to have a mean of 0 and a standard deviation of 1, and dashed portions of clustering dendrograms represent non-pregnant males.

S7 *S. scovelli* has atypical craniofacial development from early developmental stages in which cartilages can first be detected by alcian blue staining. a) Shown is a developmental series of pipefish embryos from within the brood pouch and a juvenile (one day after emergence from the pouch, at 16 days after fertilization), stained with alcian blue for cartilage and alizarin red for mineralized bone. Approximately similar developmental stages of stickleback, another percomorph, are shown. Note the upturned jaws (formed by Meckel's and palatoquadrate cartilages), the unusually curved ethmoid cartilage, and the elongating hyosymplectic cartilage in pipefish compared to stickleback, which has less derived development and morphology. b) A ventral view of pharyngeal cartilages of an 11 day old pipefish (stained with alcian and alizarin). In all panels, anterior is to the left. m, Meckel's cartilage; pq, palatoquadrate; et, ethmoid; ch, ceratohyal; hs, hyosymplectic.

S8 Maximum Likelihood (PhyML) tree from Fig. 7, including complete accession numbers for all amino acid sequences. Pastristacins are in purple. Clade support values are SH-aLRT, and number of bootstrap replicates (of 500 total) in which the clade was recovered. See Fig. 7 for context.

Additional File 1

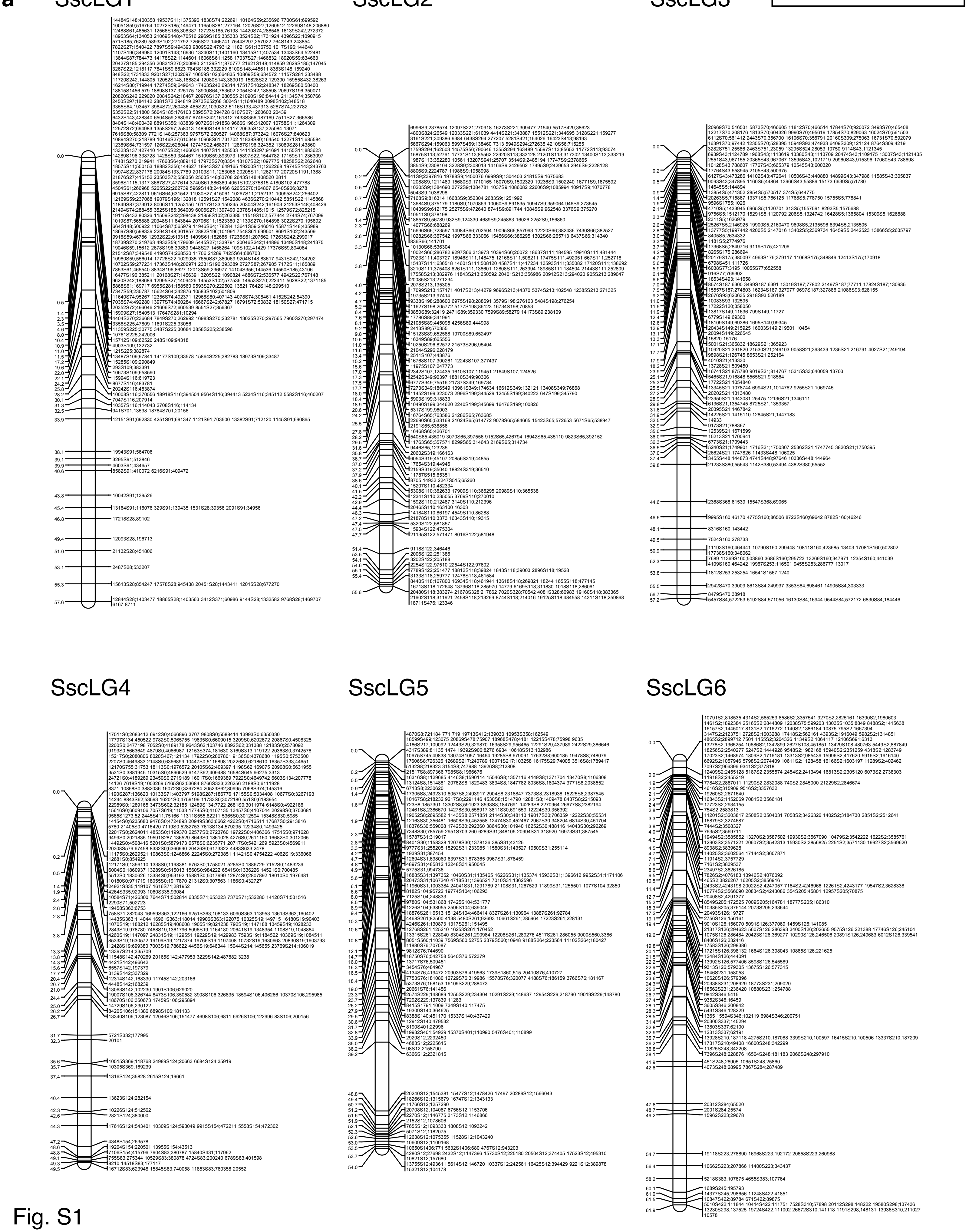

Fig. S1

a

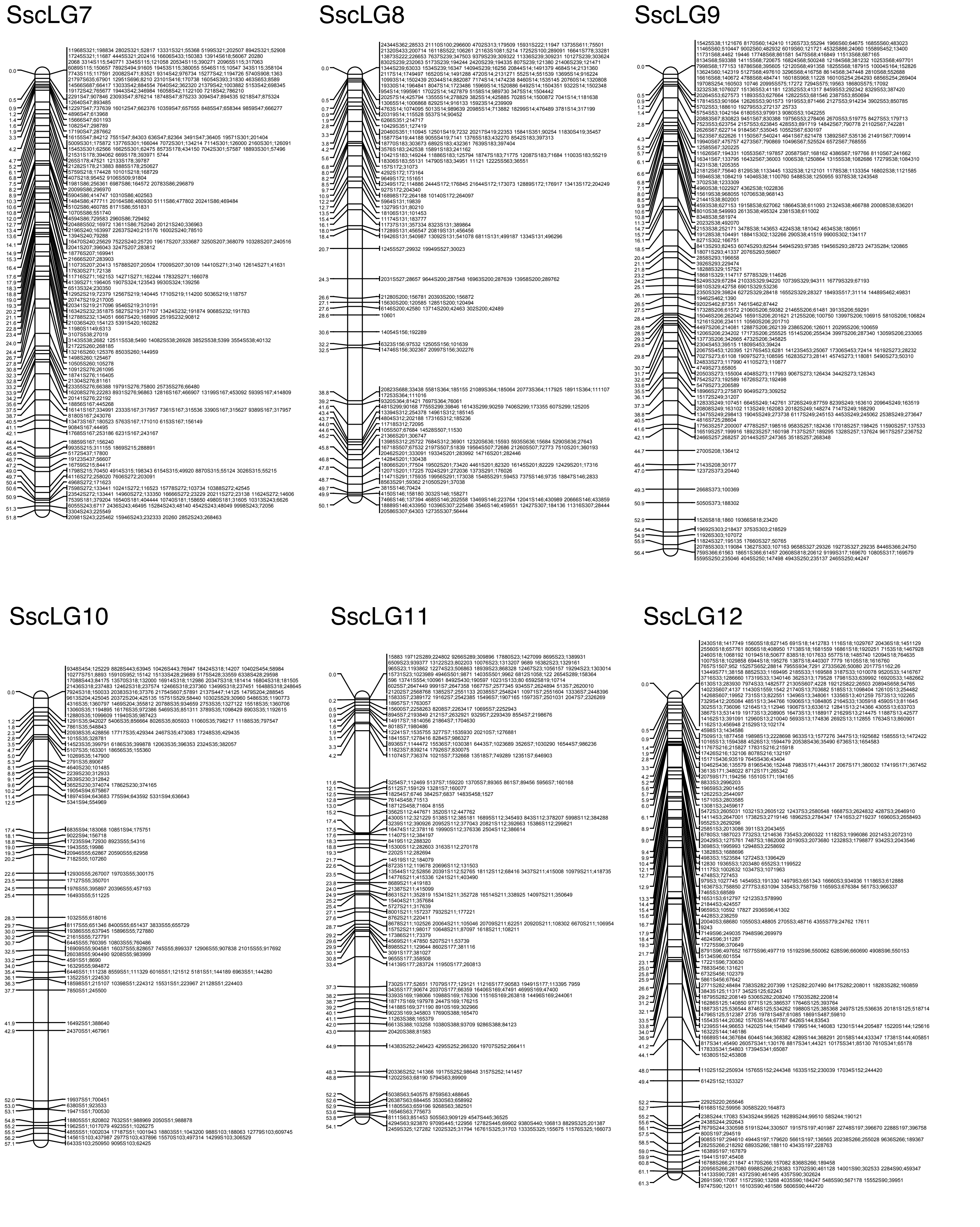

Fig. S1

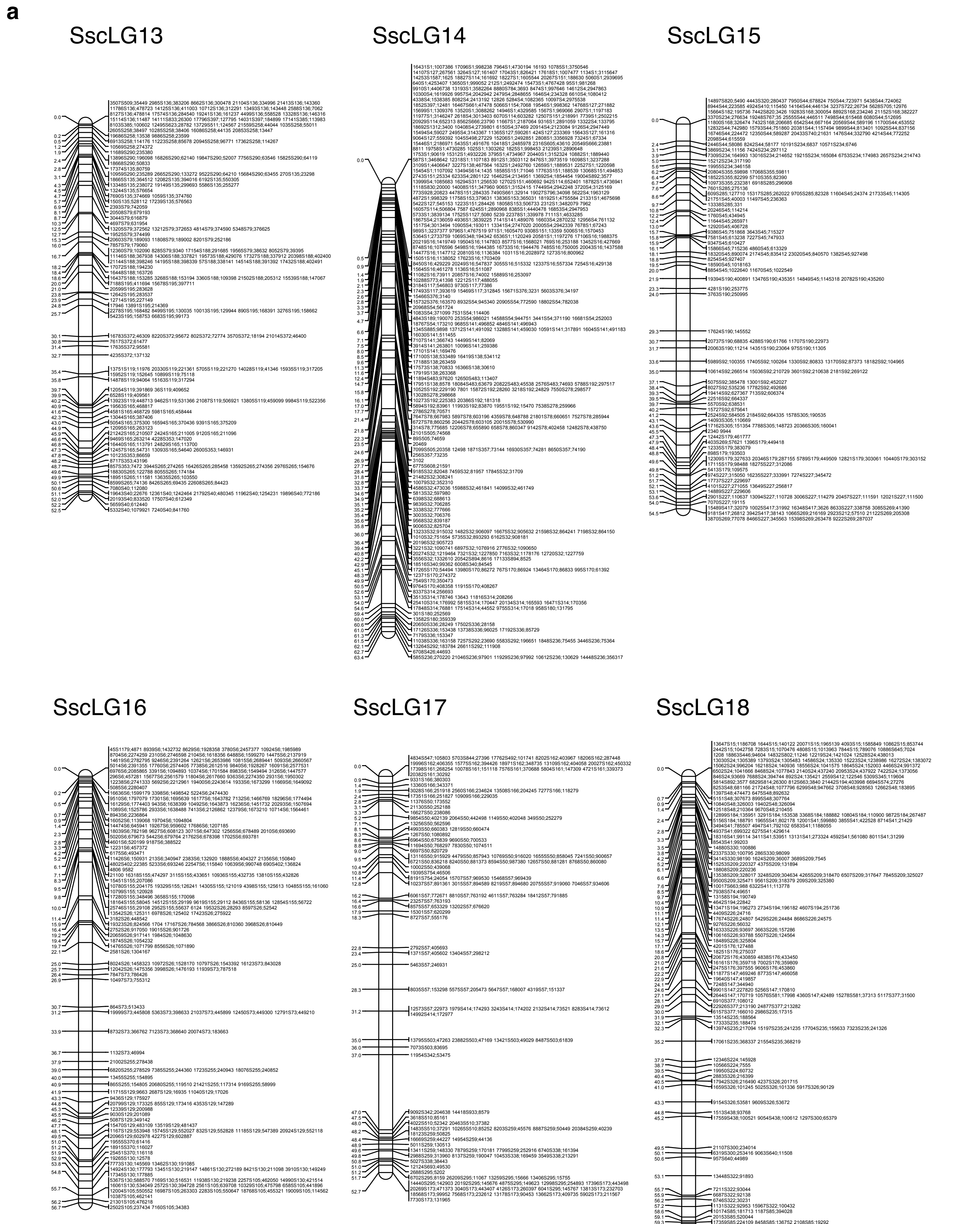

Fig. S1

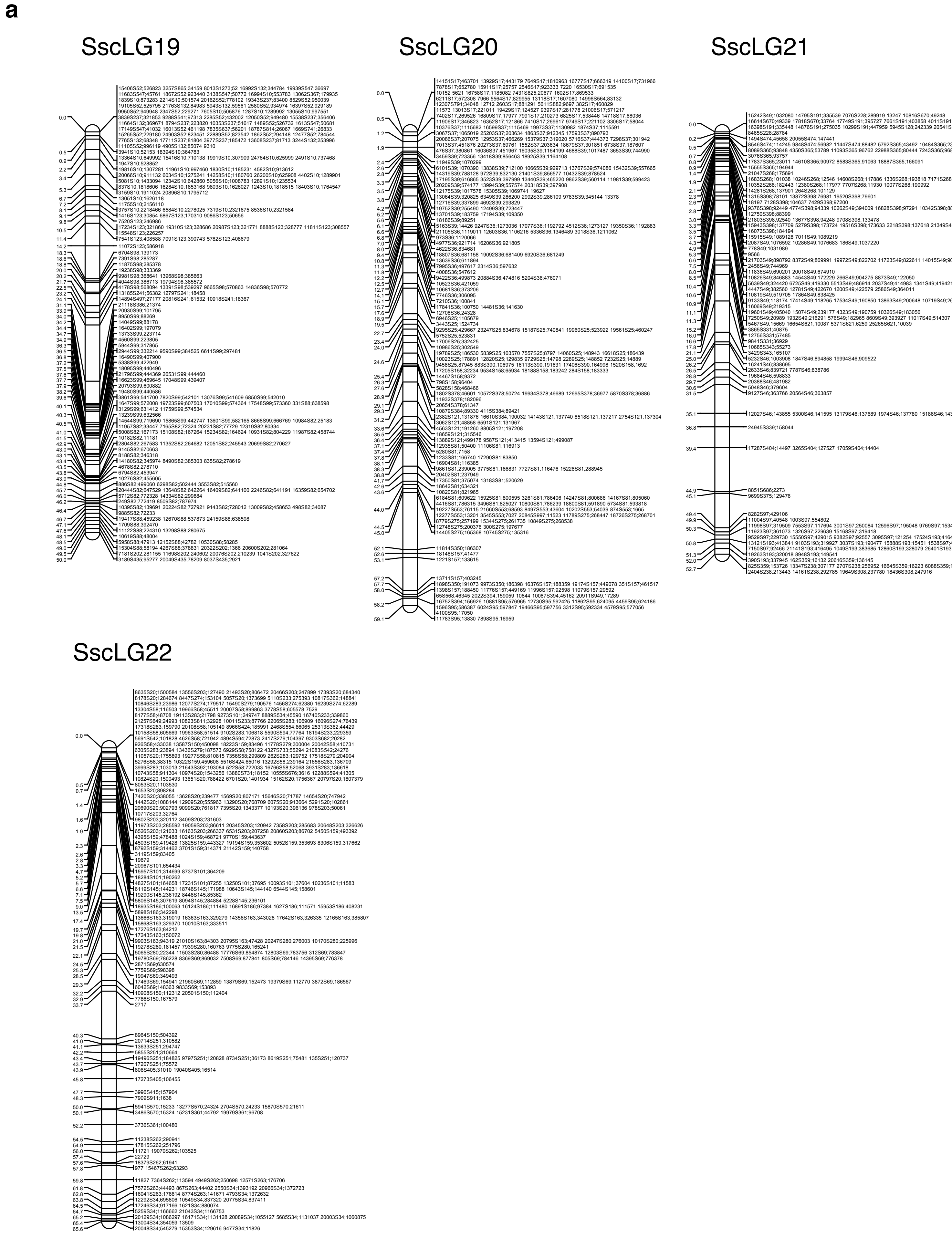

Fig. S1

b

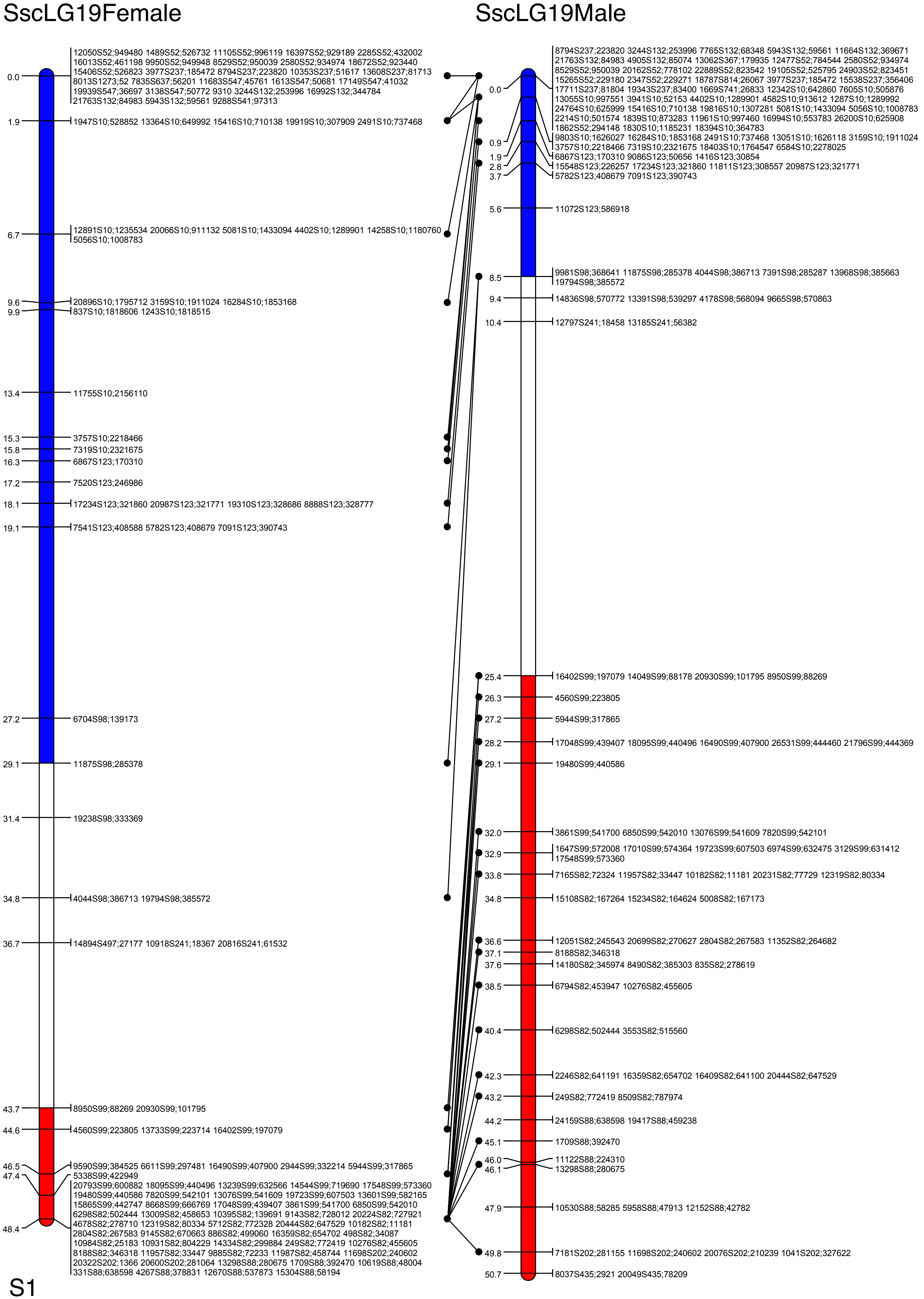
SscLG19Female
SscLG19Male
0.0 12050S52;949480 1489S52;526732 11105S52;996119 16397S52;929189 2285S52;432002
16013S52;461198 9950S52;949948 8529S52;950039 2580S52;934974 18672S52;923440
15406S52;526823 3977S237;185472 8794S237;223820 10353S237;51617 13608S237;81713
8013S1273;52 7835S637;56201 11683S547;45761 1613S547;50681 17149S547;41032
19939S547;36697 3138S547;50772 9310 3244S132;253996 16992S132;344784
21763S132;84983 5943S132;59561 9288S541;97313
1.9 1947S10;528852 13364S10;649992 15416S10;710138 19919S10;307909 2491S10;737468
6.7 12891S10;1235534 20066S10;911132 5081S10;1433094 4402S10;1289901 14258S10;1180760
5056S10;1008783
9.6 20896S10;1795712 3159S10;1911024 16284S10;1853168
9.9 837S10;1818606 1243S10;1818515
13.4 11755S10;2156110
15.3 3757S10;2218466
15.8 7319S10;2321675
16.3 6867S123;170310
17.2 7520S123;246986
18.1 17234S123;321860 20987S123;321771 19310S123;328686 8888S123;328777
19.1 7541S123;408588 5782S123;408679 7091S123;390743
27.2 6704S98;139173
29.1 11875S98;285378
31.4 19238S98;333369
34.8 4044S98;386713 19794S98;385572
36.7 14894S497;27177 10918S241;18367 20816S241;61532
43.7 8950S99;88269 20930S99;101795
44.6 4560S99;223805 13733S99;223714 16402S99;197079
46.5 9590S99;384525 6611S99;297481 16490S99;407900 2944S99;332214 5944S99;317865
47.4 5338S99;422949
48.4 20793S99;600882 18095S99;440496 13239S99;632566 14544S99;719690 17548S99;573360
19480S99;440586 7820S99;542101 13076S99;541609 19723S99;607503 13601S99;582165
15865S99;442747 8668S99;666769 17048S99;439407 3861S99;541700 6850S99;542010
6298S82;502444 13009S82;458653 10395S82;139691 9143S82;728012 20224S82;727921
4678S82;278710 12319S82;80334 5712S82;772328 20444S82;647529 10182S82;11181
2804S82;267583 9145S82;670663 886S82;499060 16359S82;654702 498S82;34087
10984S82;25183 10931S82;804229 14334S82;299884 249S82;772419 10276S82;455605
8188S82;346318 11957S82;33447 9885S82;72233 11987S82;458744 11698S202;240602
20322S202;1366 20600S202;281064 13298S88;280675 1709S88;392470 10619S88;48004
331S88;638598 4267S88;378831 12670S88;537873 15304S88;58194
0.0 8794S237;223820 3244S132;253996 7765S132;68348 5943S132;59561 11664S132;369671
21763S132;84983 4905S132;85074 13062S367;179935 12477S52;784544 2580S52;934974
8529S52;950039 20162S52;778102 22889S52;823542 19105S52;525795 24903S52;823451
15265S52;229180 2347S52;229271 18787S814;26067 3977S237;185472 15538S237;356406
17711S237;81804 19343S237;83400 1669S741;26833 12342S10;642860 7605S10;505876
13055S10;997551 3941S10;52153 4402S10;1289901 4582S10;913612 1287S10;1289992
24764S10;625999 15416S10;710138 19816S10;1307281 5081S10;1433094 5056S10;1008783
2214S10;501574 1839S10;873283 11961S10;997460 16994S10;553783 26200S10;625908
1862S52;294148 1830S10;1185231 18394S10;364783
0.9 9803S10;1626027 16284S10;1853168 2491S10;737468 13051S10;1626118 3159S10;1911024
1.9 3757S10;2218466 7319S10;2321675 18403S10;1764547 6584S10;2278025
2.8 6867S123;170310 9086S123;50656 1416S123;30854
3.7 15548S123;226257 17234S123;321860 11811S123;308557 20987S123;321771
5782S123;408679 7091S123;390743
5.6 11072S123;586918
8.5 9981S98;368641 11875S98;285378 4044S98;386713 7391S98;285287 13968S98;385663
19794S98;385572
9.4 14836S98;570772 13391S98;539297 4178S98;568094 9665S98;570863
10.4 12797S241;18458 13185S241;56382
25.4 16402S99;197079 14049S99;88178 20930S99;101795 8950S99;88269
26.3 4560S99;223805
27.2 5944S99;317865
28.2 17048S99;439407 18095S99;440496 16490S99;407900 26531S99;444460 21796S99;444369
29.1 19480S99;440586
32.0 3861S99;541700 6850S99;542010 13076S99;541609 7820S99;542101
32.9 1647S99;572008 17010S99;574364 19723S99;607503 6974S99;632475 3129S99;631412
17548S99;573360
33.8 7165S82;72324 11957S82;33447 10182S82;11181 20231S82;77729 12319S82;80334
34.8 15108S82;167264 15234S82;164624 5008S82;167173
36.6 12051S82;245543 20699S82;270627 2804S82;267583 11352S82;264682
37.1 8188S82;346318
37.6 14180S82;345974 8490S82;385303 835S82;278619
38.5 6794S82;453947 10276S82;455605
40.4 6298S82;502444 3553S82;515560
42.3 2246S82;641191 16359S82;654702 16409S82;641100 20444S82;647529
43.2 249S82;772419 8509S82;787974
44.2 24159S88;638598 19417S88;459238
45.1 1709S88;392470
46.0 11122S88;224310
46.1 13298S88;280675
47.9 10530S88;58285 5958S88;47913 12152S88;42782
49.8 7181S202;281155 11698S202;240602 20076S202;210239 1041S202;327622
50.7 8037S435;2921 20049S435;78209

Fig. S1

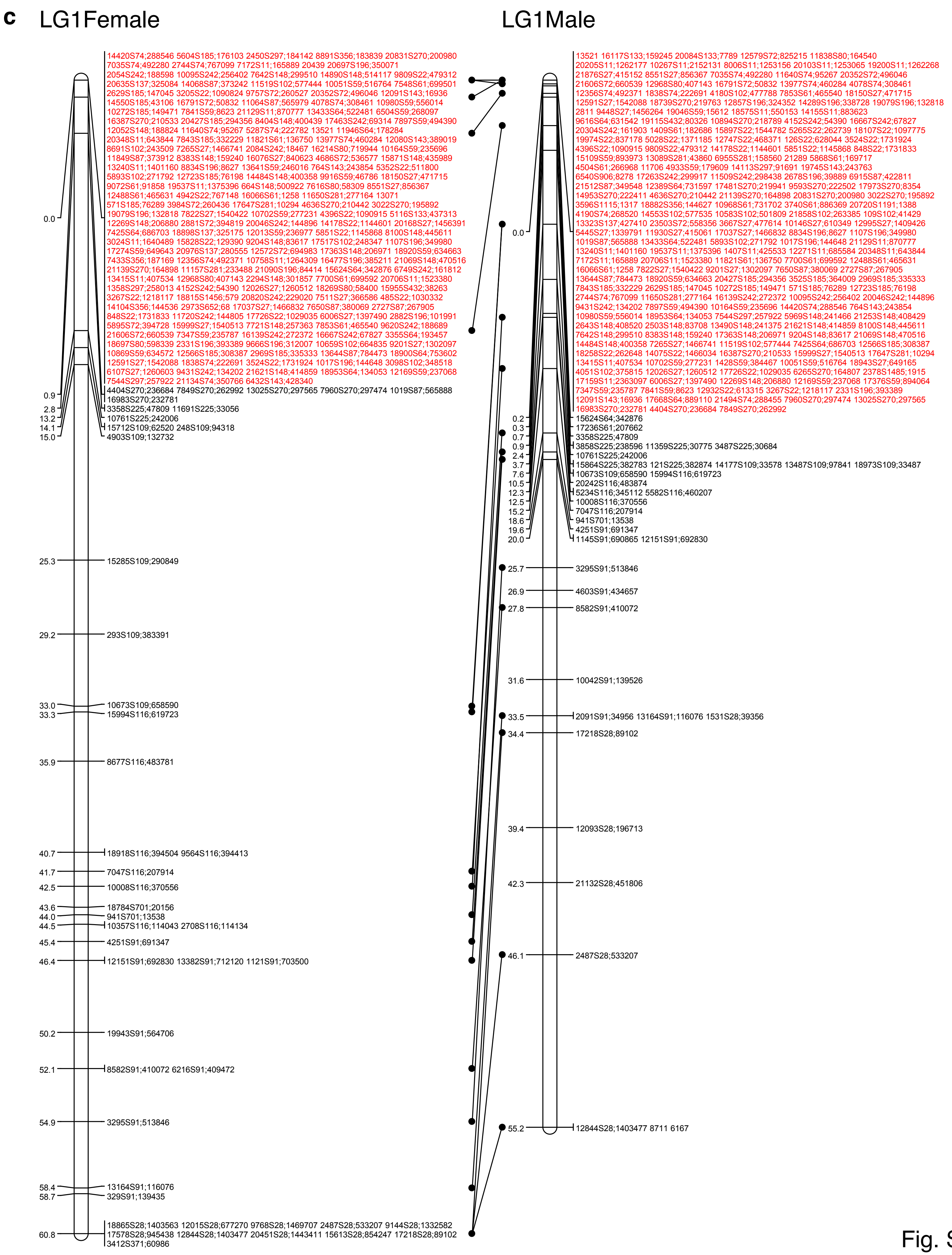
c
LG1Female
LG1Male
14420S74;288546 5604S185;176103 2450S297;184142 8891S356;183839 20831S270;200980
7035S74;492280 2744S74;767099 7172S11;165889 20439 20697S196;350071
2054S242;188598 10095S242;256402 7642S148;299510 14890S148;514117 9809S22;479312
20635S137;325084 14068S87;373242 11519S102;577444 10051S59;516764 7548S61;699501
2629S185;147045 3205S22;1090824 9757S72;260527 20352S72;496046 12091S143;16936
14550S185;43106 16791S72;50832 11064S87;565979 4078S74;308461 10980S59;556014
10272S185;149471 7841S59;8623 21129S11;870777 13433S64;522481 6504S59;268097
16387S270;210533 20427S185;294356 8404S148;400439 17463S242;69314 7897S59;494390
12052S148;188824 11640S74;95267 5287S74;222782 13521 11946S64;178284
20348S11;643844 7843S185;332229 11821S61;136750 13977S74;460284 12080S143;389019
8691S102;243509 7265S27;1466741 2084S242;18467 16214S80;719944 10164S59;235696
11849S87;373912 8383S148;159240 16076S27;840623 4686S72;536577 15871S148;435989
13240S11;1401160 8834S196;8627 13641S59;246016 764S143;243854 5352S22;511800
5893S102;271792 12723S185;76198 14484S148;400358 9916S59;46786 18150S27;471715
9072S61;91858 19537S11;1375396 664S148;500922 7616S80;58309 8551S27;856367
12488S61;465631 4942S22;767148 16066S61;1258 11650S281;277164 13071
571S185;76289 3984S72;260436 17647S281;10294 4636S270;210442 3022S270;195892
19079S196;132818 7822S27;1540422 10702S59;277231 4396S22;1090915 5116S133;437313
12269S148;206880 2881S72;394819 20046S242;144896 14178S22;1144601 20168S27;1456391
7425S64;686703 18898S137;325175 12013S59;236977 5851S22;1145868 8100S148;445611
3024S11;1640489 15828S22;129390 9204S148;83617 17517S102;248347 1107S196;349980
17274S59;649643 20976S137;280555 12572S72;694983 17363S148;206971 18920S59;634663
7433S356;187169 12356S74;492371 10758S11;1264309 16477S196;385211 21069S148;470516
21139S270;164898 11157S281;233488 21090S196;84414 15624S64;342876 6749S242;161812
13415S11;407534 12968S80;407143 2294S148;301857 7700S61;699592 20706S11;1523380
1358S297;258013 4152S242;54390 12026S27;1260512 18269S80;58400 15955S432;38263
3267S22;1218117 18815S1456;579 20820S242;229020 7511S27;366586 485S22;1030332
14104S356;144536 2973S652;68 17037S27;1466832 7650S87;380069 2727S87;267905
848S22;1731833 11720S242;144805 17726S22;1029035 6006S27;1397490 2882S196;101991
5895S72;394728 15999S27;1540513 7721S148;257363 7853S61;465540 9620S242;188689
21606S72;660539 7347S59;235787 16139S242;272372 16667S242;67827 3355S64;193457
18697S80;598339 2331S196;393389 9666S196;312007 10659S102;664835 9201S27;1302097
10869S59;634572 12566S185;308387 2969S185;335333 13644S87;784473 18900S64;753602
12591S27;1542088 1838S74;222691 3524S22;1731924 1017S196;144648 3098S102;348518
6107S27;1260603 9431S242;134202 21621S148;414859 18953S64;134053 12169S59;237068
7544S297;257922 21134S74;350766 6432S143;428340
0.0
0.9
2.8
13.2
14.1
15.0
4404S270;236684 7849S270;262992 13025S270;297565 7960S270;297474 1019S87;565888
16983S270;232781
3358S225;47809 11691S225;33056
10761S225;242006
15712S109;62520 248S109;94318
4903S109;132732
25.3 15285S109;290849
29.2 293S109;383391
33.0 10673S109;658590
33.3 15994S116;619723
35.9 8677S116;483781
40.7 18918S116;394504 9564S116;394413
41.7 7047S116;207914
42.5 10008S116;370556
43.6 18784S701;20156
44.0 941S701;13538
44.5 10357S116;114043 2708S116;114134
45.4 4251S91;691347
46.4 12151S91;692830 13382S91;712120 1121S91;703500
50.2 19943S91;564706
52.1 8582S91;410072 6216S91;409472
54.9 3295S91;513846
58.4 13164S91;116076
58.7 329S91;139435
60.8
18865S28;1403563 12015S28;677270 9768S28;1469707 2487S28;533207 9144S28;1332582
17578S28;945438 12844S28;1403477 20451S28;1443411 15613S28;854247 17218S28;89102
3412S371;60986
13521 16117S133;159245 20084S133;7789 12579S72;825215 11838S80;164540
20205S11;1262177 10267S11;2152131 8006S11;1253156 20103S11;1253065 19200S11;1262268
21876S27;415152 8551S27;856367 7035S74;492280 11640S74;95267 20352S72;496046
21606S72;660539 12968S80;407143 16791S72;50832 13977S74;460284 4078S74;308461
12356S74;492371 1838S74;222691 4180S102;477788 7853S61;465540 18150S27;471715
12591S27;1542088 18739S270;219763 12857S196;324352 14289S196;338728 19079S196;132818
2811 9448S27;1456264 19046S59;15612 18575S11;550153 14155S11;883623
9616S64;631542 19115S432;80326 10894S270;218789 4152S242;54390 16667S242;67827
20304S242;161903 1409S61;182686 15897S22;1544782 5265S22;262739 18107S22;1097775
19974S22;837178 5028S22;1371185 12747S22;468371 126S22;628044 3524S22;1731924
4396S22;1090915 9809S22;479312 14178S22;1144601 5851S22;1145868 848S22;1731833
15109S59;893973 13089S281;43860 6955S281;158560 21289 5868S61;169717
4504S61;266968 11706 4933S59;179609 14113S297;91691 19745S143;243763
6540S906;8278 17263S242;299917 11509S242;298438 2678S196;39889 6915S87;422811
21512S87;349548 12389S64;731597 17481S270;219941 9593S270;222502 17973S270;8354
14953S270;222411 4636S270;210442 21139S270;164898 20831S270;200980 3022S270;195892
3596S1115;1317 18882S356;144627 10968S61;731702 3740S61;886369 20720S1191;1388
4190S74;268520 14553S102;577535 10583S102;501809 21858S102;263385 109S102;41429
13323S137;427410 23503S72;558356 3667S27;477614 10146S27;610349 12995S27;1409426
5445S27;1339791 11930S27;415061 17037S27;1466832 8834S196;8627 1107S196;349980
1019S87;565888 13433S64;522481 5893S102;271792 1017S196;144648 21129S11;870777
13240S11;1401160 19537S11;1375396 1407S11;425533 12271S11;685584 20348S11;643844
7172S11;165889 20706S11;1523380 11821S61;136750 7700S61;699592 12488S61;465631
16066S61;1258 7822S27;1540422 9201S27;1302097 7650S87;380069 2727S87;267905
13644S87;784473 18920S59;634663 20427S185;294356 3525S185;364009 2969S185;335333
7843S185;332229 2629S185;147045 10272S185;149471 571S185;76289 12723S185;76198
2744S74;767099 11650S281;277164 16139S242;272372 10095S242;256402 20046S242;144896
9431S242;134202 7897S59;494390 10164S59;235696 14420S74;288546 764S143;243854
10980S59;556014 18953S64;134053 7544S297;257922 5969S148;241466 21253S148;408429
2643S148;408520 2503S148;83708 13490S148;241375 21621S148;414859 8100S148;445611
7642S148;299510 8383S148;159240 17363S148;206971 9204S148;83617 21069S148;470516
14484S148;400358 7265S27;1466741 11519S102;577444 7425S64;686703 12566S185;308387
18258S22;262648 14075S22;1466034 16387S270;210533 15999S27;1540513 17647S281;10294
13415S11;407534 10702S59;277231 1428S59;384467 10051S59;516764 18943S27;649165
4051S102;375815 12026S27;1260512 17726S22;1029035 6265S270;164807 2378S1485;1915
17159S11;2363097 6006S27;1397490 12269S148;206880 12169S59;237068 17376S59;894064
7347S59;235787 7841S59;8623 12932S22;613315 3267S22;1218117 2331S196;393389
12091S143;16936 17668S64;889110 21494S74;288455 7960S270;297474 13025S270;297565
16983S270;232781 4404S270;236684 7849S270;262992
0.0
0.2 15624S64;342876
0.3 17236S61;207662
0.7 3358S225;47809
0.9 3858S225;238596 11359S225;30775 3487S225;30684
2.4 10761S225;242006
3.7 15864S225;382783 121S225;382874 14177S109;33578 13487S109;97841 18973S109;33487
7.6 10673S109;658590 15994S116;619723
10.5 20242S116;483874
12.3 5234S116;345112 5582S116;460207
12.5 10008S116;370556
15.2 7047S116;207914
18.6 941S701;13538
19.6 4251S91;691347
20.0 1145S91;690865 12151S91;692830
25.7 3295S91;513846
26.9 4603S91;434657
27.8 8582S91;410072
31.6 10042S91;139526
33.5 2091S91;34956 13164S91;116076 1531S28;39356
34.4 17218S28;89102
39.4 12093S28;196713
42.3 21132S28;451806
46.1 2487S28;533207
55.2 12844S28;1403477 8711 6167

Fig. S1

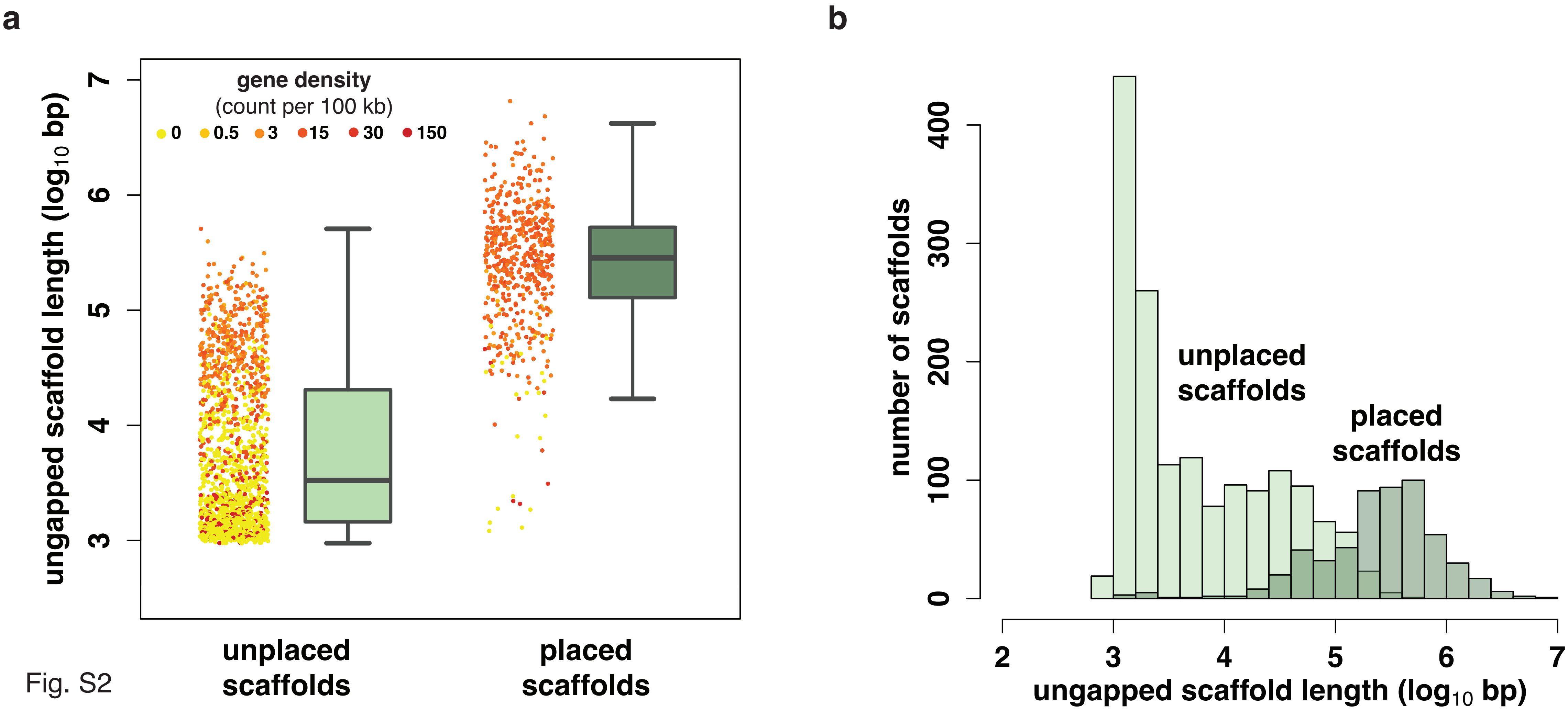
a
b
gene density
(count per 100 kb)
0
0.5
3
15
30
150
ungapped scaffold length (log10 bp)
3
4
5
6
7
unplaced
scaffolds
placed
scaffolds
number of scaffolds
0
100
200
300
400
unplaced
scaffolds
placed
scaffolds
2
3
4
5
6
7
ungapped scaffold length (log10 bp)

Fig. S2

Fig. S3

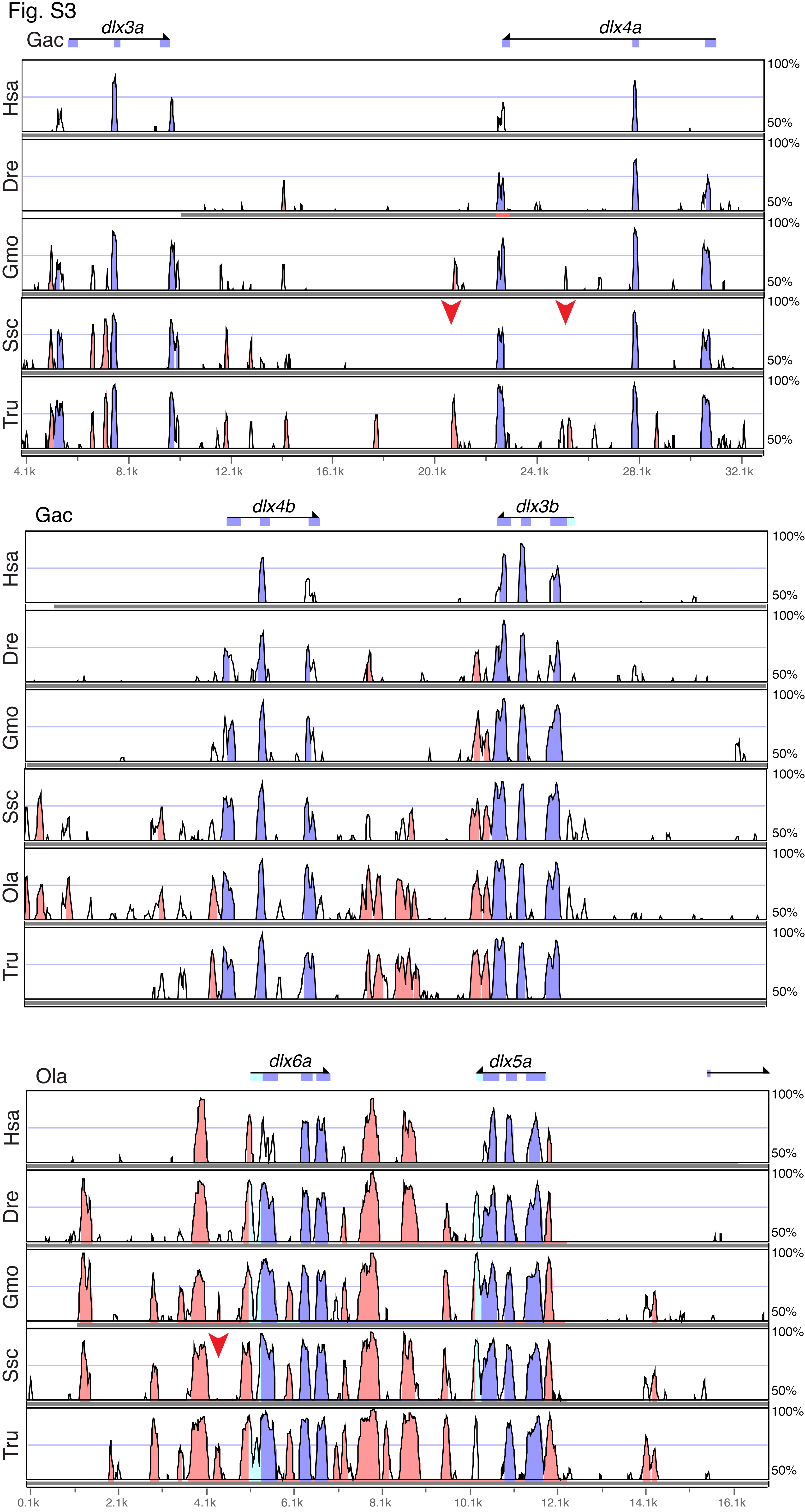

Gac
dlx3a
dlx4a
Hsa
Dre
Gmo
Ssc
Tru
100%
50%
4.1k
8.1k
12.1k
16.1k
20.1k
24.1k
28.1k
32.1k
Gac
dlx4b
dlx3b
Hsa
Dre
Gmo
Ssc
Ola
Tru
Ola
dlx6a
dlx5a
Hsa
Dre
Gmo
Ssc
Tru
0.1k
2.1k
4.1k
6.1k
8.1k
10.1k
12.1k
14.1k
16.1k

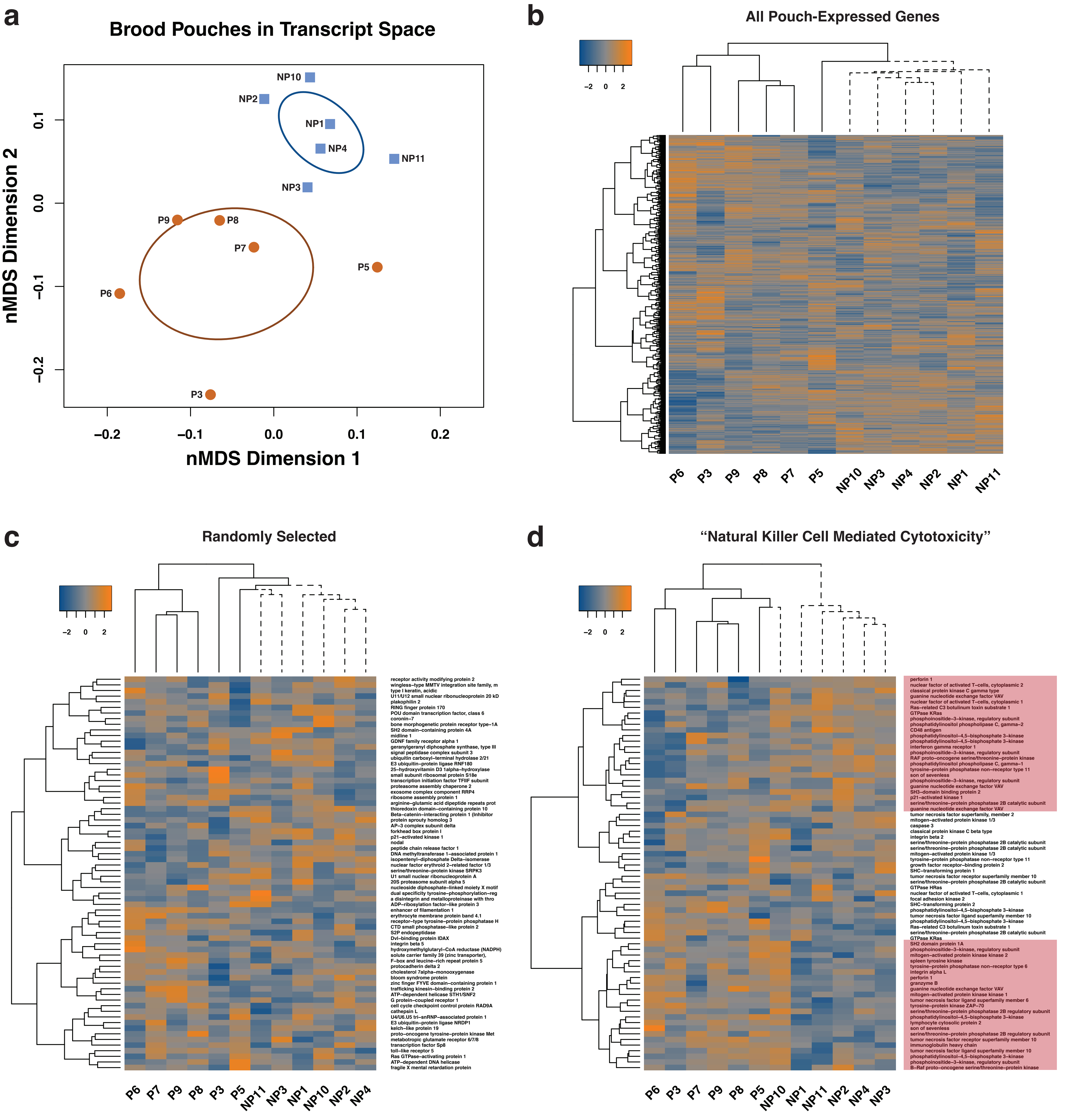
a
Brood Pouches in Transcript Space
nMDS Dimension 2
nMDS Dimension 1
0.1
0.0
−0.1
−0.2
0.2
NP10
NP2
NP1
NP4
NP11
NP3
P9
P8
P7
P5
P6
P3
b
All Pouch-Expressed Genes
−2 0 2
P6 P3 P9 P8 P7 P5 NP10 NP3 NP4 NP2 NP1 NP11
c
Randomly Selected
−2 0 2
receptor activity modifying protein 2
wingless−type MMTV integration site family, m
type I keratin, acidic
U11/U12 small nuclear ribonucleoprotein 20 kD
plakophilin 2
RING finger protein 170
POU domain transcription factor, class 6
coronin−7
bone morphogenetic protein receptor type−1A
SH2 domain−containing protein 4A
midline 1
GDNF family receptor alpha 1
geranylgeranyl diphosphate synthase, type III
signal peptidase complex subunit 3
ubiquitin carboxyl−terminal hydrolase 2/21
E3 ubiquitin−protein ligase RNF180
25−hydroxyvitamin D3 1alpha−hydroxylase
small subunit ribosomal protein S18e
transcription initiation factor TFIIF subunit
proteasome assembly chaperone 2
exosome complex component RRP4
ribosome assembly protein 1
arginine−glutamic acid dipeptide repeats prot
thioredoxin domain−containing protein 10
Beta−catenin−interacting protein 1 (Inhibitor
protein sprouty homolog 3
AP−3 complex subunit delta
forkhead box protein I
p21−activated kinase 1
nodal
peptide chain release factor 1
DNA methyltransferase 1−associated protein 1
isopentenyl−diphosphate Delta−isomerase
nuclear factor erythroid 2−related factor 1/3
serine/threonine−protein kinase SRPK3
U1 small nuclear ribonucleoprotein A
20S proteasome subunit alpha 5
nucleoside diphosphate−linked moiety X motif
dual specificity tyrosine−phosphorylation−reg
a disintegrin and metalloproteinase with thro
ADP−ribosylation factor−like protein 3
enhancer of filamentation 1
erythrocyte membrane protein band 4.1
receptor−type tyrosine−protein phosphatase H
CTD small phosphatase−like protein 2
S2P endopeptidase
Dvl−binding protein IDAX
integrin beta 5
hydroxymethylglutaryl−CoA reductase (NADPH)
solute carrier family 39 (zinc transporter),
F−box and leucine−rich repeat protein 5
protocadherin delta 2
cholesterol 7alpha−monooxygenase
bloom syndrome protein
zinc finger FYVE domain−containing protein 1
trafficking kinesin−binding protein 2
ATP−dependent helicase STH1/SNF2
G protein−coupled receptor 1
cell cycle checkpoint control protein RAD9A
cathepsin L
U4/U6.U5 tri−snRNP−associated protein 1
E3 ubiquitin−protein ligase NRDP1
kelch−like protein 19
proto−oncogene tyrosine−protein kinase Met
metabotropic glutamate receptor 6/7/8
transcription factor Sp8
toll−like receptor 5
Ras GTPase−activating protein 1
ATP−dependent DNA helicase
fragile X mental retardation protein
P6 P7 P9 P8 P3 P5 NP11 NP3 NP1 NP10 NP2 NP4
d
"Natural Killer Cell Mediated Cytotoxicity"
−2 0 2
perforin 1
nuclear factor of activated T−cells, cytoplasmic 2
classical protein kinase C gamma type
guanine nucleotide exchange factor VAV
nuclear factor of activated T−cells, cytoplasmic 1
Ras−related C3 botulinum toxin substrate 1
GTPase KRas
phosphoinositide−3−kinase, regulatory subunit
phosphatidylinositol phospholipase C, gamma−2
CD48 antigen
phosphatidylinositol−4,5−bisphosphate 3−kinase
phosphatidylinositol−4,5−bisphosphate 3−kinase
interferon gamma receptor 1
phosphoinositide−3−kinase, regulatory subunit
RAF proto−oncogene serine/threonine−protein kinase
phosphatidylinositol phospholipase C, gamma−1
tyrosine−protein phosphatase non−receptor type 11
son of sevenless
phosphoinositide−3−kinase, regulatory subunit
guanine nucleotide exchange factor VAV
SH3−domain binding protein 2
p21−activated kinase 1
serine/threonine−protein phosphatase 2B catalytic subunit
guanine nucleotide exchange factor VAV
tumor necrosis factor superfamily, member 2
mitogen−activated protein kinase 1/3
caspase 3
classical protein kinase C beta type
integrin beta 2
serine/threonine−protein phosphatase 2B catalytic subunit
serine/threonine−protein phosphatase 2B catalytic subunit
mitogen−activated protein kinase 1/3
tyrosine−protein phosphatase non−receptor type 11
growth factor receptor−binding protein 2
SHC−transforming protein 1
tumor necrosis factor receptor superfamily member 10
serine/threonine−protein phosphatase 2B catalytic subunit
GTPase HRas
nuclear factor of activated T−cells, cytoplasmic 1
focal adhesion kinase 2
SHC−transforming protein 2
phosphatidylinositol−4,5−bisphosphate 3−kinase
tumor necrosis factor ligand superfamily member 10
phosphatidylinositol−4,5−bisphosphate 3−kinase
Ras−related C3 botulinum toxin substrate 1
serine/threonine−protein phosphatase 2B catalytic subunit
GTPase KRas
SH2 domain protein 1A
phosphoinositide−3−kinase, regulatory subunit
mitogen−activated protein kinase kinase 2
spleen tyrosine kinase
tyrosine−protein phosphatase non−receptor type 6
integrin alpha L
perforin 1
granzyme B
guanine nucleotide exchange factor VAV
mitogen−activated protein kinase kinase 1
tumor necrosis factor ligand superfamily member 6
tyrosine−protein kinase ZAP−70
serine/threonine−protein phosphatase 2B regulatory subunit
phosphatidylinositol−4,5−bisphosphate 3−kinase
lymphocyte cytosolic protein 2
son of sevenless
serine/threonine−protein phosphatase 2B regulatory subunit
tumor necrosis factor receptor superfamily member 10
immunoglobulin heavy chain
tumor necrosis factor ligand superfamily member 10
phosphatidylinositol−4,5−bisphosphate 3−kinase
phosphoinositide−3−kinase, regulatory subunit
B−Raf proto−oncogene serine/threonine−protein kinase
P6 P3 P7 P9 P8 P5 NP10 NP1 NP11 NP2 NP4 NP3

Fig . S4

a

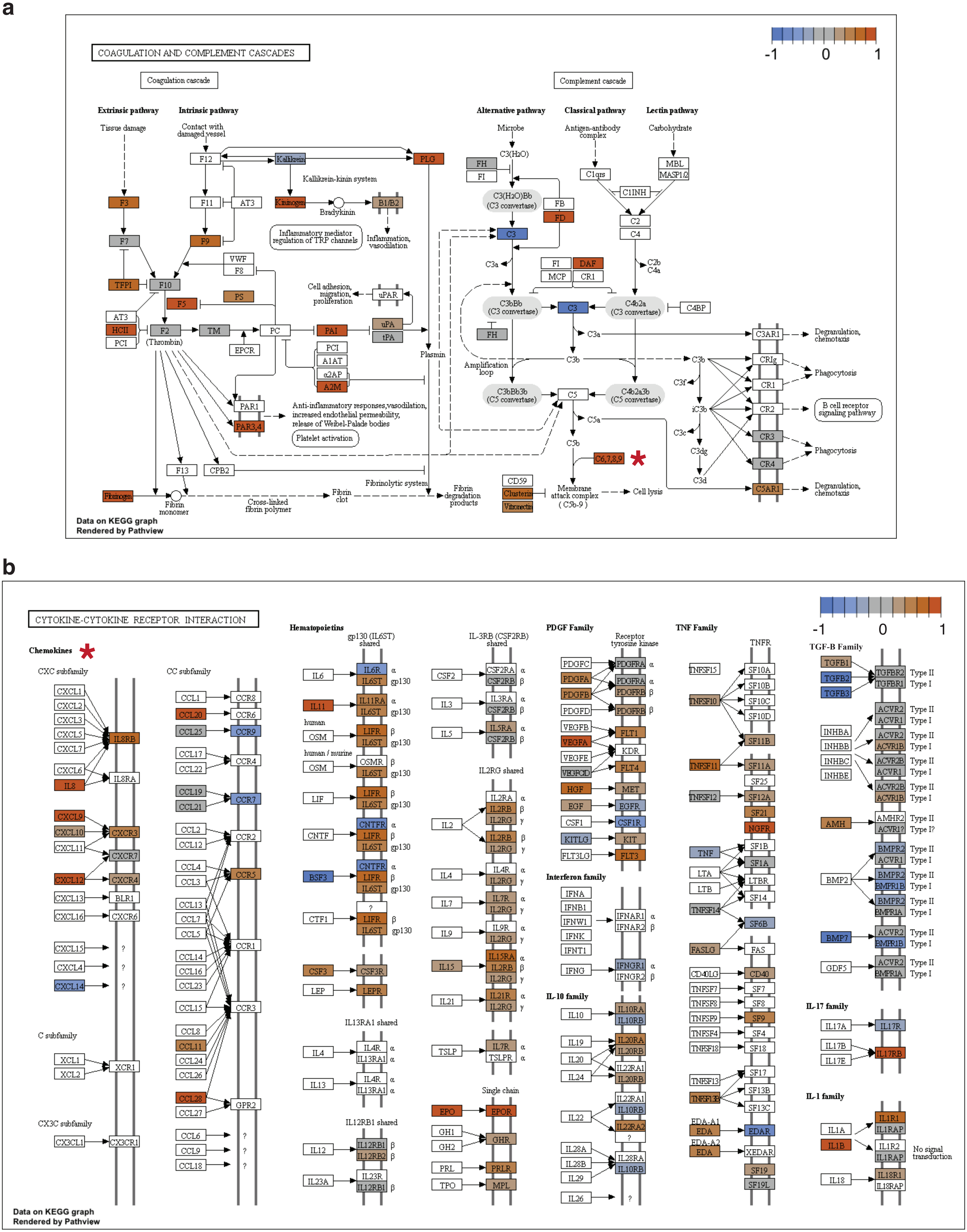
COAGULATION AND COMPLEMENT CASCADES
Coagulation cascade
Complement cascade
Extrinsic pathway
Intrinsic pathway
Alternative pathway
Classical pathway
Lectin pathway
-1
0
1
Data on KEGG graph
Rendered by Pathview
CYTOKINE-CYTOKINE RECEPTOR INTERACTION
Chemokines
CXC subfamily
CC subfamily
C subfamily
CX3C subfamily
Hematopoietins
PDGF Family
TNF Family
TGF-B Family
Interferon family
IL-10 family
IL-17 family
IL-1 family
Data on KEGG graph
Rendered by Pathview

Fig. S5

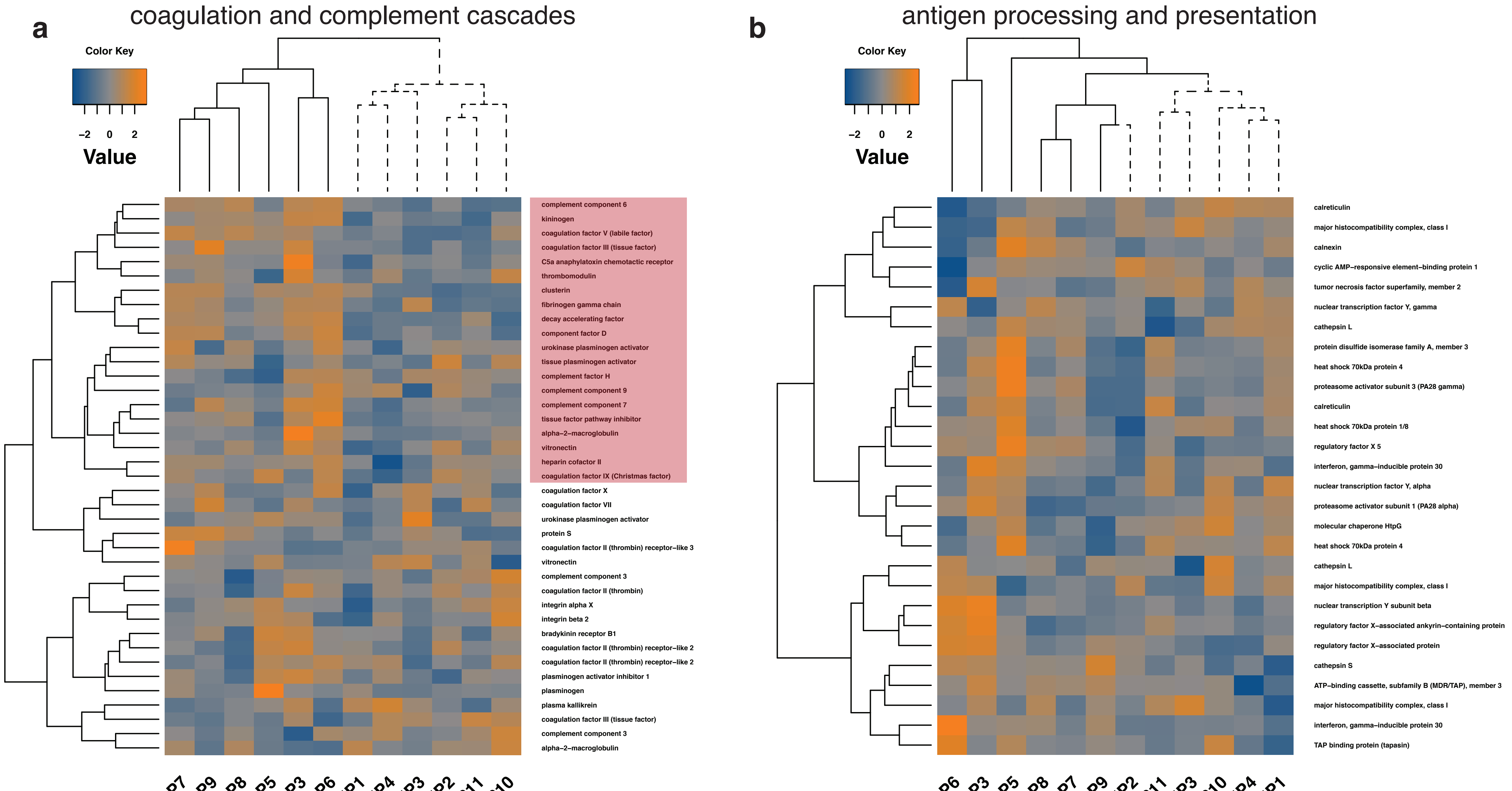
a
coagulation and complement cascades
Color Key
−2 0 2
Value
complement component 6
kininogen
coagulation factor V (labile factor)
coagulation factor III (tissue factor)
C5a anaphylatoxin chemotactic receptor
thrombomodulin
clusterin
fibrinogen gamma chain
decay accelerating factor
component factor D
urokinase plasminogen activator
tissue plasminogen activator
complement factor H
complement component 9
complement component 7
tissue factor pathway inhibitor
alpha−2−macroglobulin
vitronectin
heparin cofactor II
coagulation factor IX (Christmas factor)
coagulation factor X
coagulation factor VII
urokinase plasminogen activator
protein S
coagulation factor II (thrombin) receptor−like 3
vitronectin
complement component 3
coagulation factor II (thrombin)
integrin alpha X
integrin beta 2
bradykinin receptor B1
coagulation factor II (thrombin) receptor−like 2
coagulation factor II (thrombin) receptor−like 2
plasminogen activator inhibitor 1
plasminogen
plasma kallikrein
coagulation factor III (tissue factor)
complement component 3
alpha−2−macroglobulin
P7 P9 P8 P5 P3 P6 NP1 NP4 NP3 NP2 NP11 NP10
b
antigen processing and presentation
Color Key
−2 0 2
Value
calreticulin
major histocompatibility complex, class I
calnexin
cyclic AMP−responsive element−binding protein 1
tumor necrosis factor superfamily, member 2
nuclear transcription factor Y, gamma
cathepsin L
protein disulfide isomerase family A, member 3
heat shock 70kDa protein 4
proteasome activator subunit 3 (PA28 gamma)
calreticulin
heat shock 70kDa protein 1/8
regulatory factor X 5
interferon, gamma−inducible protein 30
nuclear transcription factor Y, alpha
proteasome activator subunit 1 (PA28 alpha)
molecular chaperone HtpG
heat shock 70kDa protein 4
cathepsin L
major histocompatibility complex, class I
nuclear transcription Y subunit beta
regulatory factor X−associated ankyrin−containing protein
regulatory factor X−associated protein
cathepsin S
ATP−binding cassette, subfamily B (MDR/TAP), member 3
major histocompatibility complex, class I
interferon, gamma−inducible protein 30
TAP binding protein (tapasin)
P6 P3 P5 P8 P7 P9 NP2 NP11 NP3 NP10 NP4 NP1

Fig. S6

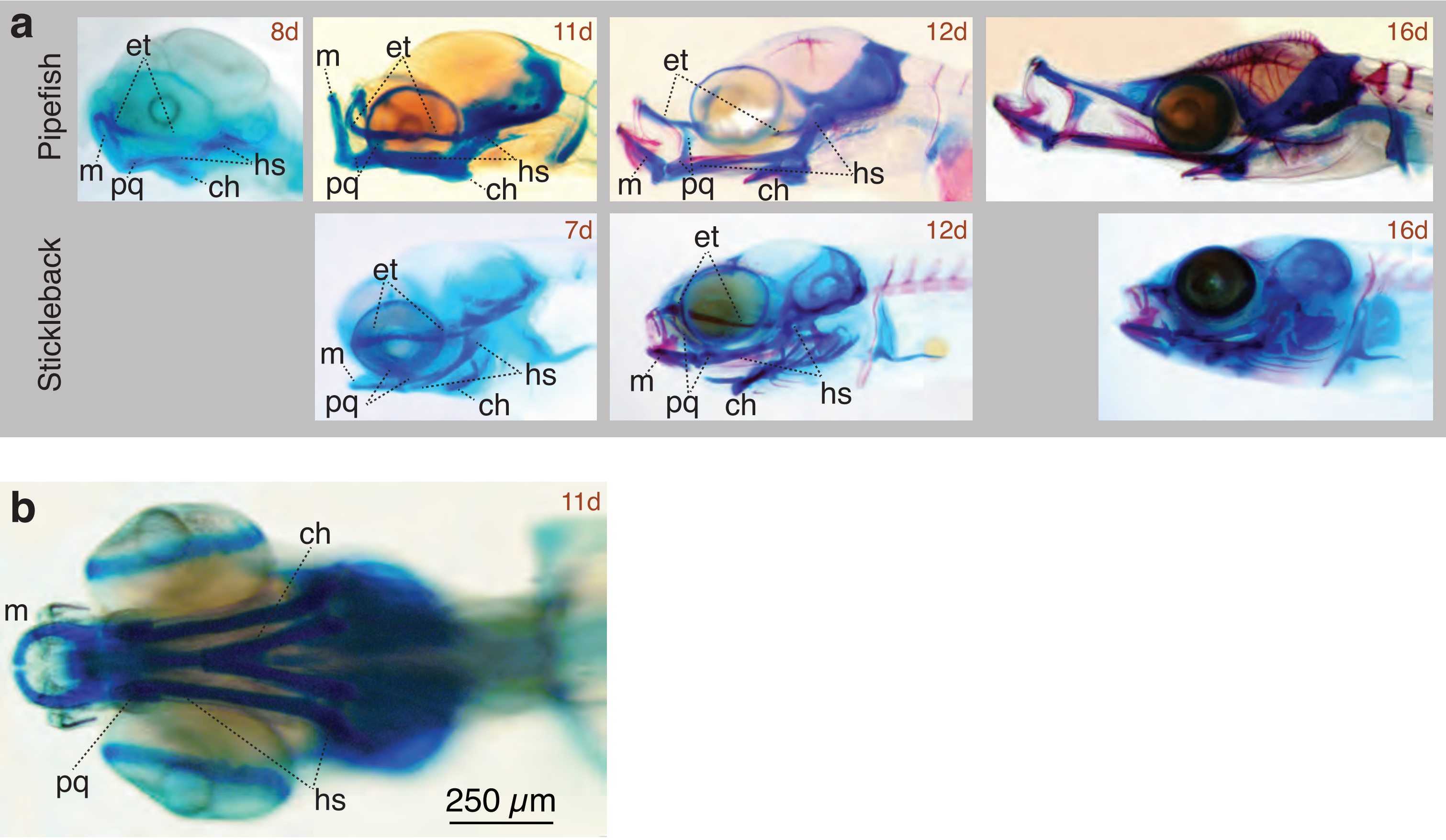
a
Pipefish
Stickleback
8d
11d
12d
16d
7d
12d
16d
et
m
pq
ch
hs
b
11d
ch
m
pq
hs
250 μm

Fig. S7

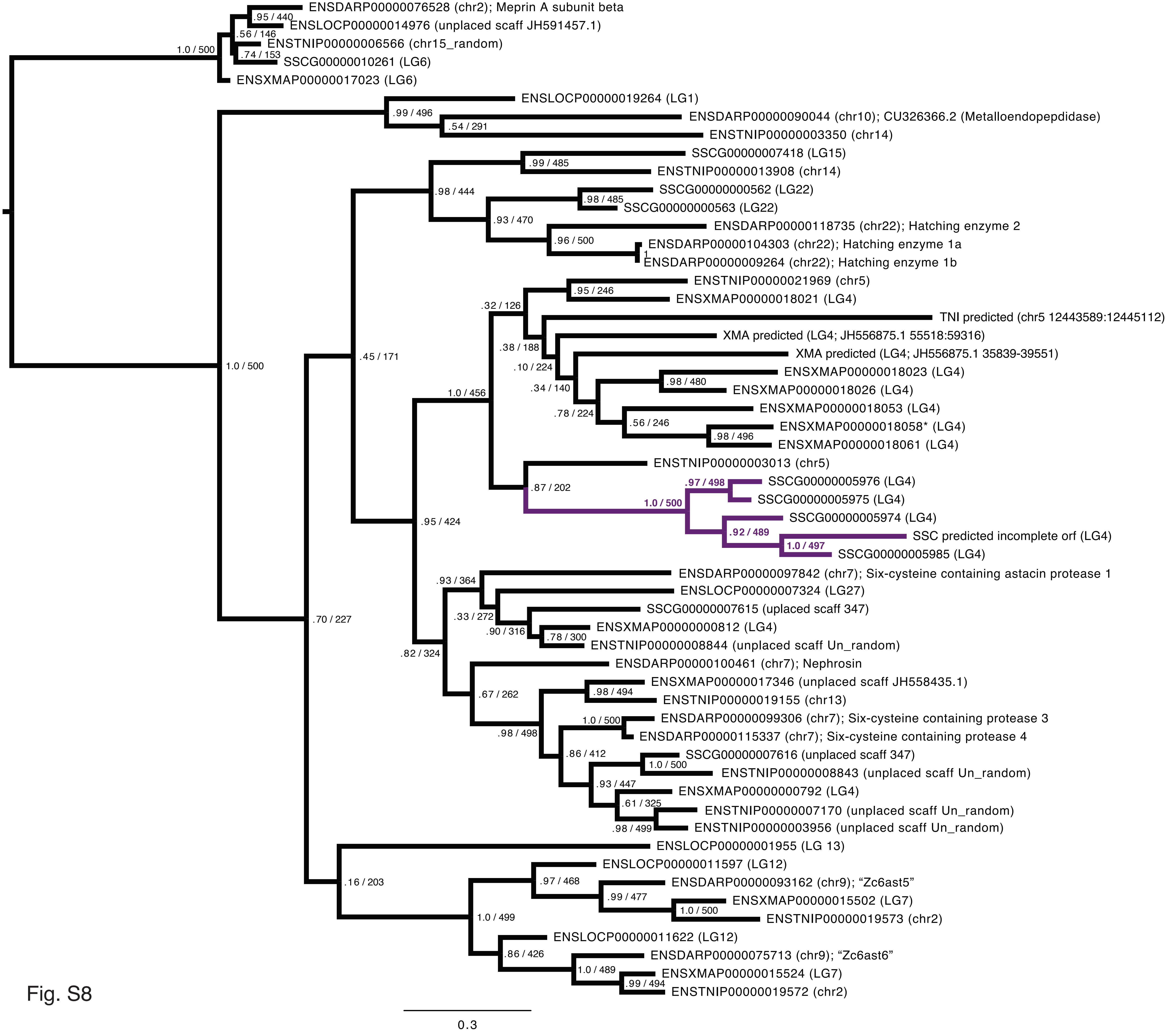
ENSDARP00000076528 (chr2); Meprin A subunit beta
.95 / 440
ENSLOCP00000014976 (unplaced scaff JH591457.1)
.56 / 146
ENSTNIP00000006566 (chr15_random)
1.0 / 500
.74 / 153
SSCG00000010261 (LG6)
ENSXMAP00000017023 (LG6)
ENSLOCP00000019264 (LG1)
.99 / 496
ENSDARP00000090044 (chr10); CU326366.2 (Metalloendopepdidase)
.54 / 291
ENSTNIP00000003350 (chr14)
SSCG00000007418 (LG15)
.99 / 485
ENSTNIP00000013908 (chr14)
SSCG00000000562 (LG22)
.98 / 444
.98 / 485
SSCG00000000563 (LG22)
.93 / 470
ENSDARP00000118735 (chr22); Hatching enzyme 2
.96 / 500
ENSDARP00000104303 (chr22); Hatching enzyme 1a
1
ENSDARP00000009264 (chr22); Hatching enzyme 1b
ENSTNIP00000021969 (chr5)
.95 / 246
ENSXMAP00000018021 (LG4)
.32 / 126
TNI predicted (chr5 12443589:12445112)
XMA predicted (LG4; JH556875.1 55518:59316)
.38 / 188
.45 / 171
XMA predicted (LG4; JH556875.1 35839-39551)
.10 / 224
ENSXMAP00000018023 (LG4)
.34 / 140
.98 / 480
ENSXMAP00000018026 (LG4)
1.0 / 500
1.0 / 456
ENSXMAP00000018053 (LG4)
.78 / 224
.56 / 246
ENSXMAP00000018058* (LG4)
.98 / 496
ENSXMAP00000018061 (LG4)
ENSTNIP00000003013 (chr5)
.97 / 498
SSCG00000005976 (LG4)
.87 / 202
1.0 / 500
SSCG00000005975 (LG4)
.95 / 424
SSCG00000005974 (LG4)
.92 / 489
SSC predicted incomplete orf (LG4)
1.0 / 497
SSCG00000005985 (LG4)
ENSDARP00000097842 (chr7); Six-cysteine containing astacin protease 1
.93 / 364
ENSLOCP00000007324 (LG27)
SSCG00000007615 (uplaced scaff 347)
.33 / 272
ENSXMAP00000000812 (LG4)
.70 / 227
.90 / 316
.78 / 300
ENSTNIP00000008844 (unplaced scaff Un_random)
.82 / 324
ENSDARP00000100461 (chr7); Nephrosin
ENSXMAP00000017346 (unplaced scaff JH558435.1)
.67 / 262
.98 / 494
ENSTNIP00000019155 (chr13)
1.0 / 500
ENSDARP00000099306 (chr7); Six-cysteine containing protease 3
.98 / 498
ENSDARP00000115337 (chr7); Six-cysteine containing protease 4
.86 / 412
SSCG00000007616 (unplaced scaff 347)
1.0 / 500
ENSTNIP00000008843 (unplaced scaff Un_random)
.93 / 447
ENSXMAP00000000792 (LG4)
.61 / 325
ENSTNIP00000007170 (unplaced scaff Un_random)
.98 / 499
ENSTNIP00000003956 (unplaced scaff Un_random)
ENSLOCP00000001955 (LG 13)
ENSLOCP00000011597 (LG12)
.16 / 203
.97 / 468
ENSDARP00000093162 (chr9); “Zc6ast5”
.99 / 477
ENSXMAP00000015502 (LG7)
1.0 / 500
1.0 / 499
ENSTNIP00000019573 (chr2)
ENSLOCP00000011622 (LG12)
.86 / 426
ENSDARP00000075713 (chr9); “Zc6ast6”
1.0 / 489
ENSXMAP00000015524 (LG7)
.99 / 494
ENSTNIP00000019572 (chr2)
0.3

Fig. S8
