# Supplementary material for: The genome of the Gulf pipefish enables understanding of evolutionary innovations
Source: Genome Biol. 2016 Dec 20;17:258. doi: 10.1186/s13059-016-1126-6 (PMC5168715; doi:10.1186/s13059-016-1126-6)
Supplement: Additional file 3: — This doc file is a comprehensive summary of Illumina data used for the Gulf pipefish genome project. A diverse collection of short-read sequencing data was used to understand the genome of S. scovelli. Included are descriptions of library types, raw read counts, and analyses in which the different libraries were used. (DOCX 90 kb) [file 13059_2016_1126_MOESM3_ESM.docx]

**Additional File 3**: A comprehensive summary of Illumina data used for the Gulf pipefish genome project

| Library Name | Library Type | Insert Size  (approx.) | Read Length | Raw Reads  (x 10^6^) | Analysis |
| --- | --- | --- | --- | --- | --- |
| WGS_180 | shotgun gDNA | 180 nt | 100 nt PE | 344.3 | APLG Assembly  SGA Assembly |
| MP_5k | mate-pair | 2.5-5 kb | 100 nt PE | 94.6 | APLG Assembly  SGA Assembly |
| MP_10k | mate-pair | 5-10 kb | 100 nt PE | 42.3 | APLG Assembly  SGA Assembly |
| MP_15k | mate-pair | 11-15 kb | 100 nt PE | 6.8 | APLG Assembly  SGA Assembly |
| MP_8k | mate-pair | 2-8 kb | 100 nt PE | 1.4 | Velvet Assembly  SGA Assembly |
| WGS_470 shotgun gDNA 470 nt | | | 80 nt PE | 26.9 | Velvet Assembly  SGA Assembly |
|  |  |  | 100 nt PE | 110.8 |  |
|  |  |  | 120 nt PE | 27.4 |  |
| RNA_emb mRNA-seq 225 nt | | | 60 nt PE | 30.2 | Embryo Transcriptome  Annotation |
|  |  |  | 100 nt PE | 140.0 |  |
| RNA_pouch  (12 total) | mRNA-seq | 225 nt | 100 nt PE | 279.6  (mean = 23.3) | Pouch Transcriptome  Annotation  Diff. Expression |
| RAD_parents  (2 total) | RAD-seq | 500 nt | 100 nt SE  75 nt PE | 13.8  (mean = 6.9) | Genetic Map |
| RAD_F1s  (108 total) | RAD-seq | 500 nt | 100 nt SE | 353.2  (mean = 3.3) | Genetic Map |
